# Quantifying proton-induced membrane polarization in single biomimetic giant vesicles

**DOI:** 10.1016/j.bpj.2022.05.041

**Published:** 2022-05-28

**Authors:** Ran Tivony, Marcus Fletcher, Ulrich F. Keyser

**Affiliations:** 1 Cavendish Laboratory, University of Cambridge, Cambridge, United Kingdom

## Abstract

Proton gradients are utilized by cells to power the transport activity of many membrane proteins. Synthetic cells, such as proteo-giant unilamellar vesicles, offer an advanced approach for studying the functionality of membrane proteins in isolation. However, understanding of protein-based transport in vitro requires accurate measurements of proton flux and its accompanying electrochemical gradient across the lipid bilayer. We present an approach to directly quantify the flux of protons across single cell-sized lipid vesicles under modulated electrochemical gradients. Our measurements reveal the corresponding association between proton permeation and transmembrane potential development and its relation to the chemical nature of the conjugated anion (base). In the case of formic acid, we showed that, out of the total amount of permeated protons, a fraction of ≈0.2 traverse the lipid bilayer as H^+^, with the rest (≈0.8) in the form of a neutral acid. For strong acids (HCl or HNO_3_), proton permeation was governed by translocation of H^+^. Accordingly, a larger proton motive force (pmf) was generated for strong acids (pmf=14.2 mV) relative to formic acid (pmf=1.3 mV). We anticipate that our approach will guide the development of protein-based transport driven by proton gradient in artificial cell models and enable a deeper understanding of how vital acids, such as fatty acids, amino acids, bile acids, and carboxylic acid-containing drugs, traverse the lipid bilayer.

## Significance

Cells generate and harness electrochemical proton gradients to drive the transport activity of various membrane proteins, from ATP synthase to bacterial efflux pumps. To elucidate the functionality of these proteins in artificial cell models, it is essential to resolve the correlation between electrochemical gradient evolution and proton permeation across the lipid bilayer. For this purpose, we directly quantified proton flux under modulated electrochemical gradients across single cell-sized lipid vesicles. We were able to differentiate between protons that translocate as ions or in the form of an uncharged acid and determine the corresponding development of transmembrane potential. Our results indicate a suitable approach for elucidating protein-based transport in synthetic cell systems.

## Introduction

Ion flux across biological membranes is vital for regulating cellular processes and maintaining homeostasis (1). In cells and organelles, ions are translocated primarily through channels and pumps (2) but can also passively leak through the phospholipid bilayer (3). In fact, these two permeation routes are coupled and operate synchronously to modulate the electrochemical gradients that propel many essential cellular activities such as transport of nutrients, organelle acidification, and transmission of electrical signals (4). Therefore, accurate quantification of ionic movement and corresponding electrochemical gradients across the lipid bilayer is important for discriminating protein-mediated transport from passive permeation in transport studies using artificial cell systems (5, 6, 7, 8, 9).

Synthetic lipid vesicles (liposomes), spherical compartments enclosed by a phospholipid bilayer, have proven a successful biomimetic system for scrutinizing the movement of ions through biological membranes (10, 11, 12). Over the last decades, vesicle-based transport studies have provided a wealth of information about the passive leakage of ions and their permeability coefficient (P) (13, 14, 15, 16, 17, 18), a fundamental property that quantifies the rate at which a solute crosses the membrane. A prominent example is the finding that protons can pass through the lipid bilayer with exceptionally high rates (${\text{P}}_{{\text{H}}^{+}}\approx 10^{-4}$ cm/s), several orders of magnitude larger than other biologically relevant monovalent ions such as Na^+^ and K^+^ (19,20). To account for their anomalously high permeability, Paula et al. showed that proton leakage involves their diffusion (i.e., hopping) through hydrated defects in the membrane (i.e., the transient pore mechanism) rather than by a simple diffusion across the nonpolar hydrocarbons of the lipid bilayer (i.e., the solubility-diffusion mechanism) (16). Nonetheless, since the obtained proton permeation coefficients (${\text{P}}_{{\text{H}}^{+}}$) were found to differ by several orders of magnitude between various studies (18,19,21, 22, 23, 24), the origin of proton leakage is still under intense scrutiny (25,26). One source for discrepancies in ${\text{P}}_{{\text{H}}^{+}}$ arises from the fact that the majority of ion permeation studies to date were performed using an ensemble of vesicles, producing averaged permeability coefficients that only reflect the collective property of the examined dispersion.

Fundamentally, protons are utilized by cells to drive the biochemical activity of various membrane proteins, from ATP synthase to bacterial efflux pumps, through generation of electrochemical gradients (or a proton motive force [pmf]). Artificial cell models, such as proteoliposomes, offer an advanced approach to study membrane proteins in isolation (27). However, interpretation of protein functionality in synthetic cells relies on accurate determination of proton fluxes and resultant electrochemical gradients (8). For instance, it is generally accepted that weak organic acids diffuse through the lipid bilayer in their neutral form (HA) following protonation of the conjugated anion (A^−^) close to the membrane surface (28, 29, 30). In the case of strong inorganic acids, however, it is believed that only dissociated protons (H^+^) cross the membrane (8,19,31), indicating that the generation of electrochemical gradients due to proton permeation varies with the chemical nature of their conjugated anion. As such, quantifying the fraction of permeated charged and uncharged species (i.e., HA and H^+^), for a given acid, is essential for resolving the level of membrane polarization and, subsequently, the correlation between proton motive force (pmf) and protein activity in vitro.

Unlike bulk permeability assays, in which the properties of the liposome population vary widely and cannot be precisely measured (12), single-vesicle analysis provides information at a higher level of detail, enabling the determination of ionic flux and permeability coefficient for each vesicle separately (8,31,32). Giant unilamellar vesicles (GUVs), cell-sized lipid vesicles, provide an even higher degree of precision, as they can be easily visualized under a light microscope while settled at the bottom of an observation chamber or trapped in a microfluidic chip (33). Likewise, their biologically relevant size makes GUVs an appropriate biomimetic model for studying transport phenomena in cells and organelles (5,6,9,34, 35, 36). Nevertheless, despite being extensively used as an established synthetic model for investigating membrane phenomena, there have been only limited attempts to employ GUVs for studying passive ion permeation (34, 35, 36).

Here, we describe an approach to resolve the correlation between the time-dependent flux and electrochemical gradients of protons across the lipid bilayer of cell-sized vesicles. Through this approach, we were able to determine the flux at different proton gradients and quantify the permeability coefficient and corresponding development of transmembrane potential (i.e., membrane polarization). By analyzing the gradient-dependent flux of protons, we showed that in the case of strong acids, such as HCl or HNO_3_, proton permeation is governed by translocation of proton ions (H^+^) under our experimental conditions. However, in the case of a weak organic acid such as formic acid, protons flow through the lipid bilayer both in the form of an undissociated acid (HA) and as H^+^, generating lower diffusion potentials. We further evaluated the relative flux of each permeated form and found that, in the case of formic acid, only a small fraction (≈0.2) leaks as proton ions while the rest (≈0.8) leaks as a neutral acid. Consequently, we evaluated the maximal pmf generated for each type of acid.

## Materials and methods

### Materials

1,2-Dioleoyl-*sn*-glycero-3-phosphocholine (DOPC), 1,2-dioleoyl-*sn*-glycero-3-phospho-(1′-rac-glycerol) (sodium salt) (DOPG), 1,2-dioleoyl-*sn*-glycero-3-phosphoethanolamine-*N*-(lissamine rhodamine B sulfonyl) (ammonium salt) (18:1 Liss Rhod PE), and 1,2-dioleoyl-*sn*-glycero-3-phosphoethanolamine-*N*-(7-nitro-2-1,3-benzoxadiazol-4-yl) (ammonium salt) (18:1 NBD PE) were purchased from Avanti Polar Lipids (Alabaster, AL) as powder and dissolved in chloroform to a final concentration of 100 mg/mL (DOPC and DOPG) and 1 mg/mL (Liss Rhod PE and NBD PE). 8-Hydroxypyrene-1,3,6-trisulfonic acid trisodium salt (HPTS) was purchased from Merck (Kenilworth, NJ) and used as received. 1-Octanol was purchased from Sigma (St. Louis, MO) and used as received. Polydimethylsiloxane (PDMS) Sylgard 184 was purchased from Dow Corning (Midland, MI) and used as received.

### Fabrication of microfluidic device

The PDMS microfluidic devices were fabricated using photolithography and soft lithography, as previously described elsewhere (37). The master mold for the octanol-assisted liposome assembly (OLA) design was prepared by spin-coating a thin layer of SU-8 2025 photoresist (Chestech, Rugby, UK) on a 4-inch silicon wafer (University Wafer, South Boston, MA), to generate a silicon master with feature heights around 20 μm. The wafer was then soft-baked (65°C for 1 min and at 95°C for 6 min), and the structures (designed in AutoCAD) were imprinted on the substrate with UV light using a tabletop laser direct imaging system (LPKF ProtoLaser; LPKF, Garbsen, Germany). The formed structures were then post-baked (1 min at 65°C and for 6 min at 95°C) and developed with propylene glycol monomethyl ether acetate. Finally, the wafer was hard-baked for 10 min at 120°C.

The PDMS chips were prepared by casting a degassed liquid PDMS (9:1 ratio with a curing agent) into the mold and then curing it for at least 2 h at 60°C. The hardened PDMS was taken off the mold and bonded to a PDMS-coated coverslip after oxygen plasma treatment for 10 s (100 W plasma power, 25 sccm, plasma etcher; Diener Electric, Ebhausen, Germany).

### Preparation of giant unilamellar vesicles

GUVs were prepared using a microfluidic-based technique, OLA (38), which enables high-throughput and controlled production of monodispersed GUVs in various buffers and an excellent encapsulation efficiency. In brief, OLA uses flow focusing in a six-way junction to form GUVs through dewetting of double-emulsion (w/o/w) capsules composed of an inner aqueous phase (IA), a lipid-octanol oil phase (LO), and an outer aqueous phase (OA). Operation of the microfluidic device is achieved via a pressure-driven pump by which flow rates of all three phases (IA, LO, and OA) can be tuned and monitored in real time. For all experiments, the base solution used for the IA and OA phases (prepared in Milli-Q water) consisted of buffer A: 10 mM HEPES and 200 mM sucrose, titrated with 1 M NaOH to reach a final pH value of 7.6. The IA (buffer A) also contained the membrane-impermeable pH-sensitive dye HPTS (pyranine, 10 μM), and the OA phase (pH 7.6) also included 50 mg/mL Kolliphor P-188 (Sigma-Aldrich, Gillingham, UK). The LO phase comprised 4 μL of a lipid stock solution (100 mg/mL DOPC:DOPG in ethanol; 3:1 v/v ratio), 1 μL of a fluorescent-lipid solution (1 mg/mL Liss Rhod PE in chloroform), and 95 μL of 1-octanol.

Electroformed GUVs were prepared using a Nanion Vesicle Prep Pro setup (Nanion, Munich, Germany). DOPC, DOPG, and Liss Rhod PE were dissolved in chloroform in a ratio of 3:1:0.01 w/w. The lipid mixture at 5 mg/ml (80 μl) was spin-coated on the conducting surface of an indium tin oxide (ITO)-coated glass slide (Nanion/VisionTek, Chester, UK). The chloroform was evaporated for 3 h in a desiccator, then 700 μL of buffer A (see above) with 10 *μ*M HPTS was deposited within an O-ring chamber, which was then sealed with another ITO-coated slide. The electroformation protocol was carried out at 37°C and proceeded in three steps: 1) the AC voltage increased linearly from 0 to 3 V (peak to peak [p-p]) at 10 Hz over 40 min; 2) the voltage stayed at 3 V (p-p) and 10 Hz for 60 min; 3) the voltage decreased linearly to 0 V at 10 Hz over 20 min.

### Data acquisition and visualization of proton permeation

Images and videos of GUVs were acquired by an inverted laser scanning confocal microscope (Olympus Fluoview FV1200, IX83; Olympus, Tokyo, Japan), using a 40× oil immersion objective (Olympus UPlanFL N). The lumen (HPTS labeled) and membrane (Liss Rhod-PE labeled) of the vesicles were visualized following their excitation with a 488 nm argon laser and 559 nm LED laser, respectively (the vesicle membrane was imaged for image analysis purposes—see below). Prior to their imaging, the GUVs were settled on a PDMS-coated glass slide (0.13 mm) in a custom-designed PDMS observation chamber by adding 20 *μ*L of vesicles dispersion (without pyranine in the external solution) to 40 *μ*L of buffer A′, an isosmotic glucose solution containing 10 mM HEPES, 200 mM glucose, and 15 *μ*M pyranine (pH 7.6). The final pyranine concentration in the external solution was 10 *μ*M.

Permeation experiments were performed after GUVs were left to sediment in the observation chamber for at least 1–2 h, and all measurements were repeated at least three times using different vesicle samples. Proton permeation was initiated by pipetting 1–1.5 *μ*L of an acid solution (1 wt% HCl, 2 wt% HNO_3_, 2 wt% HCOOH) to the vesicles sample (60 *μ*L). To capture the initial extravesicular and lumenal pyranine intensities, time-lapse imaging of the vesicles was performed ≈1 min prior to the addition of acid. All videos were acquired at a frame rate of either 0.2 frames/s, for HCl and HNO_3,_ or 0.3 frames/s, for HCOOH.

### Image analysis

All images and videos were analyzed using a custom Python script (Zenodo: https://doi.org/10.5281/zenodo.6344232) that automatically detects, tracks, and measures the fluorescence intensity of HPTS within the GUVs lumen. Extraction of vesicles from confocal images (or image stack) was performed through detecting the Liss Rhod PE signal of the GUV equator using the Hough Circle Transform algorithm. Since the sedimented GUVs sometimes move laterally within the image frame boundaries, to ensure a consistent analysis of the same vesicle throughout the duration of the experiment all detected GUVs were tracked and a unique ID was assigned for each one of them. Vesicle tracking was performed by calculating the minimal Euclidean distance between a vesicle’s centroid and all other detected centroids in previous frames. Finally, the GUV radius and lumenal HPTS intensity were measured for every image in the acquired measurement video.

## Results and discussion

### Resolving proton permeation across single cell-sized vesicles

**Figure 1:**
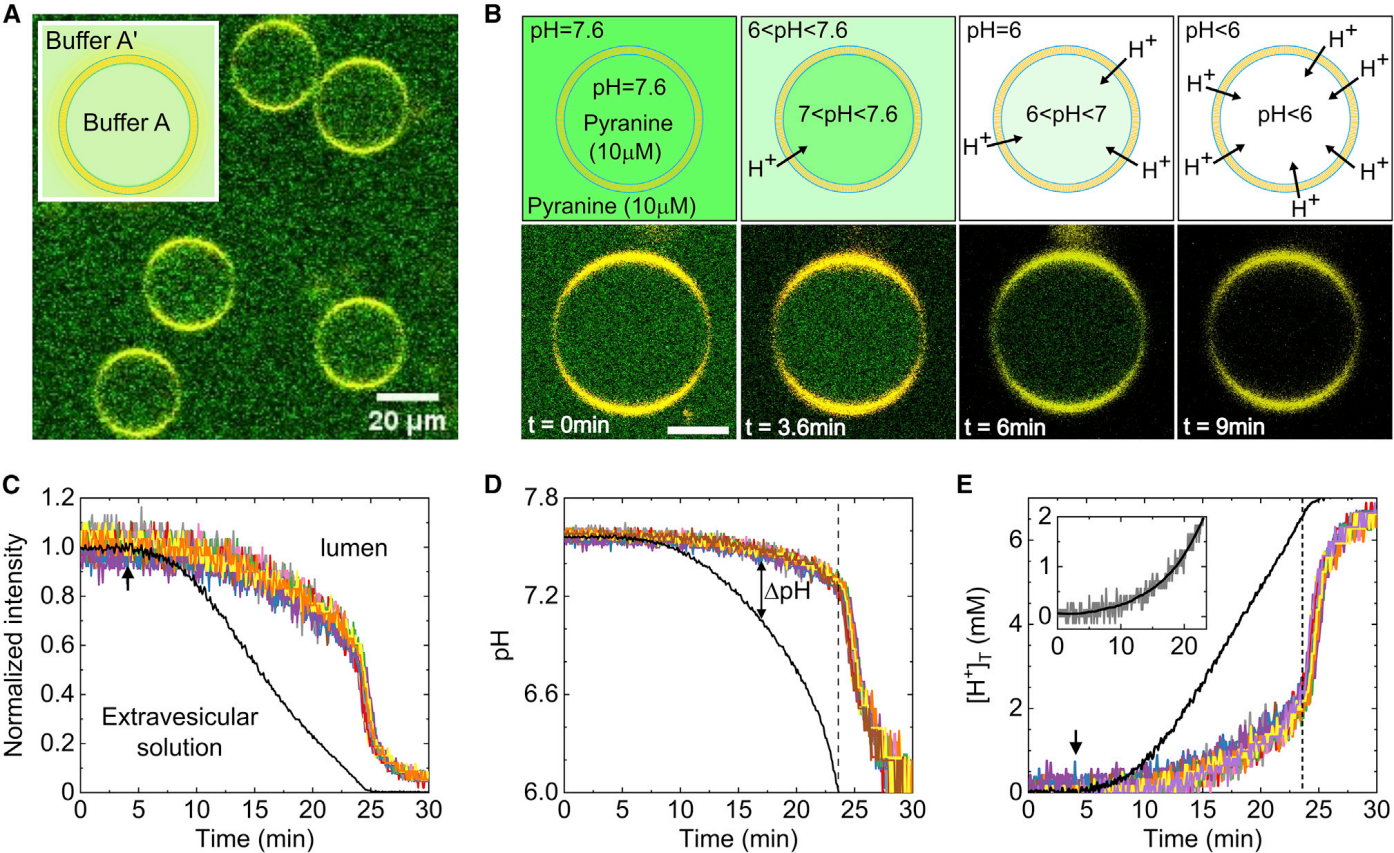
Time-resolved analysis of proton permeation across single cell-sized vesicles following the addition of HCl. (*A*) Confocal image of DOPC:DOPG (3:1) GUVs loaded with buffer A (10 mM HEPES, 200 mM sucrose, and 10 *μ*M pyranine; pH 7.6) settled at the bottom of an observation chamber in a buffer A′ (10 mM HEPES, 200 mM glucose, and 10 *μ*M pyranine; pH 7.6). (*B*) Upper: schematic showing the basic concept of the ion permeation assay. A minute volume of acid solution is added to the external buffer and allowed to diffuse toward the resting vesicles, generating a gradually increased pH gradient across their membrane. Lower: proton permeation across a single cell-sized vesicle as illustrated by the change of background and lumenal pyranine intensity. (*C*) Analysis of proton permeation from pyranine fluorescence intensity at the extravesicular solution (*black line*) and lumen of single GUVs (*colored lines*). Permeation measurements were conducted at 21°C. (*D*) Quantification of background and lumenal pH values corresponding to the intensity curves shown in Fig. 2*C*. The black dashed line signifies the point beyond which the background pH cannot be resolved due to complete quenching of pyranine intensity. (*E*) Total concentration of permeated protons (*colored lines*) and protons outside the vesicles (*black line*). The black dashed line indicates the relevant time frame for flux analysis. Inset: the black line shows an example of a smoothed concentration curve from which the flux is determined. To see this figure in color, go online.

Accurate analysis of ion permeation rates from flux measurements across liposomes requires a knowledge of lumenal and extravesicular ion concentrations as well as of their volume and surface area. To this end, we used cell-sized lipid vesicles that can be easily resolved under a light microscope and designed an approach that simultaneously monitors the concentration of ions on both sides of the membrane. Negatively charged GUVs, encompassing a sucrose-containing HEPES buffer solution (buffer A, pH 7.6) and pyranine, a pH-dependent dye that has a negligible membrane permeability during the time course of our experiments, were prepared through a microfluidic-based technique, octanol-assisted liposome assembly (OLA) (37,38). We precisely determined the temporal evolution of proton concentration on both sides of the vesicle membrane by settling GUVs at the bottom of an observation chamber in an isosmotic glucose-containing HEPES buffer (buffer A′, pH 7.6) with a pyranine concentration that matched their interior content (Fig. 1 *A*). Next, a 1–1.5 *μ*L aliquot of acid solution was added to the surrounding solution (see materials and methods) and allowed to diffuse toward and, subsequently, into the vesicles, as schematically illustrated in the upper panel of Fig. 1 *B*.

Through this approach, the fluorescence intensity outside and inside a single GUV can be gradually changed over time (Fig. 1 *B*, *lower panel*) so that the arrival time of protons and their permeation at small concentration gradients can be precisely determined (Fig. 1 *C*), unlike in acid-pulse assays which assume an instantaneous acidification of the external solution (18). We also confirmed the absence of pyranine photobleaching during the measurement period (Fig. S1) and that its fluorescence is altered due to proton permeation and not by leaking through the GUVs membrane (Fig. S2). Furthermore, analysis of extravesicular (*black curve*) and lumenal (*colored curves*) pH values (Figs. 1 *D* and S3) reveals that, following the addition of acid, protons progressively accumulate at the exterior side of the vesicle lipid bilayer while leaking through it at a much slower rate, indicating that their permeation is not rate limited by diffusion through the external unstirred layer (3). Likewise, the fast diffusion time of protons across the internal unstirred layer $t=r^{2}/6{\text{D}}_{{\text{H}}^{+}}\approx 2\;$ ms (i.e., from the membrane to the center of the GUV), relative to the rate of data acquisition in our experiments (see materials and methods), ensures that their concentration inside the vesicles is uniform and measured accurately, where r=10 *μ*m is the vesicle’s radius and ${\text{D}}_{{\text{H}}^{+}}=9.3\times 10^{-5}$ cm^2^/s is the diffusion coefficient of protons (39). Accordingly, the pH difference (ΔpH) across the GUV membrane can be determined for every time frame, allowing us to explore the passive leakage of protons over a range of concentration gradients. In addition, the generation and dissipation rate of ΔpH can be modified by the initial amount of added acid so that the flux of protons can be investigated over a wider range of concentration gradients (Fig. S4). We note, however, that since pyranine is completely quenched below pH = 6 (Fig. S3), proton permeation can only be resolved for 6 < pH < 7.6, as indicated by the black dashed line and visualized in Fig. 1 *B*.

### Proton permeation under modulated electrochemical gradients

In biological systems, the ionic flux is regulated by the concentration and electric potential gradients across the membrane J(ΔC,Δψ) and is typically described by the Goldman-Hodgkin-Katz (GHK) flux density equation (1):

JH+=PH+×ΔψFRT×[H+]i−[H+]oe−ΔψFRT1−e−ΔψFRT,

where ${\left[{\text{H}}^{+}\right]}_{\text{i}}=10^{-{\text{pH}}_{\text{i}}}/1000$ and ${\left[{\text{H}}^{+}\right]}_{\text{o}}=10^{-{\text{pH}}_{\text{o}}}/1000$ are the concentration of ${\text{H}}^{+}$ (mol/cm^3^) inside and outside the vesicle, respectively, ${\text{P}}_{{\text{H}}^{+}}$ (cm/s) is the proton permeability coefficient, and *F*, *R*, and *T* have their usual meaning. In the absence of a transmembrane potential, however, the flux density (mol/cm^2^/s) depends only on ΔC and is defined by Fick’s law:

$${\text{J}}_{{\text{H}}^{+}}={\text{P}}_{{\text{H}}^{+}}\times \left({\left[{\text{H}}^{+}\right]}_{\text{o}}-{\left[{\text{H}}^{+}\right]}_{\text{i}}\right).$$

Remarkably, it has been shown that the conductance (and, thus, the flux) of protons across various lipid bilayers is nearly independent of pH, suggesting that their permeability coefficient is pH dependent (19,24,40). Therefore, it is of interest to examine the relation between the flux and concentration gradients in our system in order to extract the permeability coefficient and unravel the underlying pathway through which protons passively leak through the lipid bilayer.

By quantifying the concentration of protons in the external solution ${\left[{\text{H}}^{+}\right]}_{\text{o}}$ from measured pH_o_ values (Fig. 1 *D*, *black curve*) we are able to determine the concentration gradient of protons $\Delta \left[{\text{H}}^{+}\right]={\left[{\text{H}}^{+}\right]}_{\text{o}}-{\left[{\text{H}}^{+}\right]}_{\text{i}}$ from the time of proton arrival (*black arrow* in Fig. 1 *E*) up to a period where their exterior concentration can no longer be resolved (*black dashed line* in Fig. 1 *E*). The inward flux ${\text{J}}_{{\text{H}}^{+}}$ (per unit area) of protons was quantified through ${\text{J}}_{{\text{H}}^{+}}=-\frac{\text{d}{\left[{\text{H}}^{+}\right]}_{\text{T}}^{\text{in}}}{\text{dt}}\left(\frac{\text{V}}{\text{A}}\right)$, where *V* is the vesicle volume, *A* is the membrane surface area, and $\text{d}{\left[{\text{H}}^{+}\right]}_{\text{T}}^{\text{in}}$ is the change of the total concentration of permeated protons in any time interval d*t*. We analyzed the time-dependant concentration of translocated protons ${\left[{\text{H}}^{+}\right]}_{\text{T}}^{\text{in}}\left(\text{t}\right)={\text{dpH}}_{\text{i}}\left(\text{t}\right)\times \text{β}\left(\text{pH}\right)$, shown in Fig. 1 *E* (*colored curves*), through separately measuring the pH-dependent buffer capacity β(pH) (Fig. S5) and the variation of lumenal pH (see materials and methods) between consecutive time frames ${\text{dpH}}_{\text{i}}\left(\text{t}\right)={\text{pH}}_{\text{i}}\left(\text{t}\right)-{\text{pH}}_{\text{i}}\left(\text{t}+1\right)$. Subsequently, the influx of ${\text{H}}^{+}$ is found by determining the volume and surface area of each GUV and from the change of ${\left[{\text{H}}^{+}\right]}_{\text{T}}^{\text{in}}$ with respect to time $\left(\text{d}{\left[{\text{H}}^{+}\right]}_{\text{T}}^{\text{in}}/\text{dt}\right)$, as determined by smoothing the ${\left[{\text{H}}^{+}\right]}_{\text{T}}^{\text{in}}$ curves (Fig. 1 *E*, *inset*).

**Figure 2:**
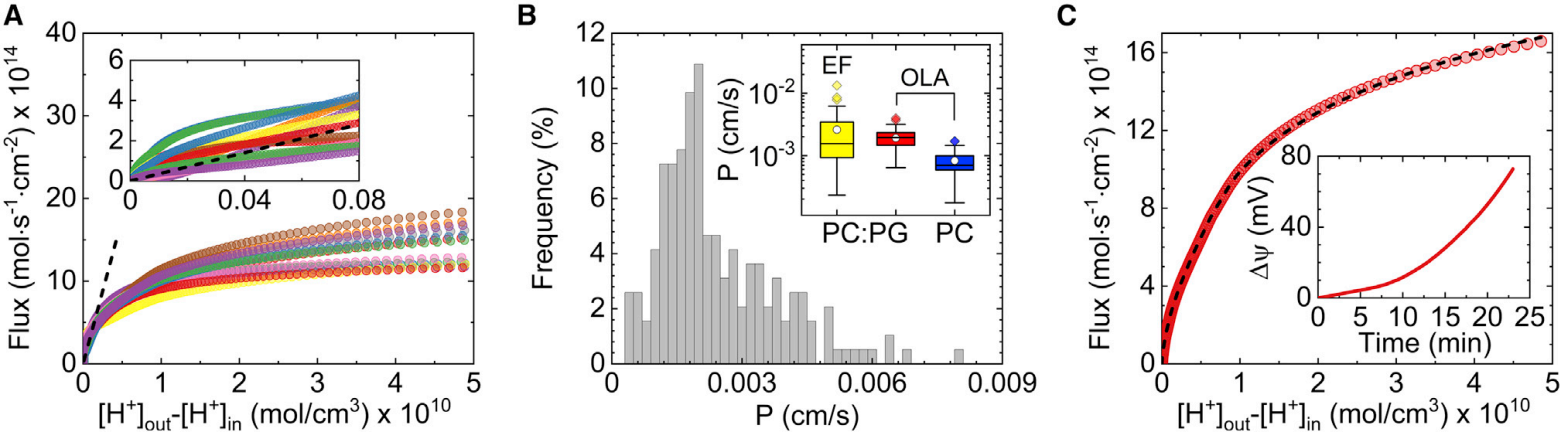
Quantification of permeation coefficient and transmembrane potential from flux profiles following the addition of HCl. (*A*) Single-vesicle-level measurement (at 21°C) of proton flux at different concentration gradients, $\Delta \left[{\text{H}}^{+}\right]={\left[{\text{H}}^{+}\right]}_{\text{o}}-{\left[{\text{H}}^{+}\right]}_{\text{i}}$. Inset: a magnified view of the flux profiles at small $\Delta \left[{\text{H}}^{+}\right]$ values color coded to match the curves in the main figure. The black dashed line shown in the inset is the concatenate fit of the flux equation ${\text{J}}_{{\text{H}}^{+}}=\text{P}\Delta \left[{\text{H}}^{+}\right]$ to the plotted flux profiles, as obtained by linear regression. The same linear fit is also shown in the main figure as a black dashed line. (*B*) Frequency distribution of measured permeability coefficients as obtained for negatively charged DOPC:DOPG (3:1 w/w) GUVs (*n* = 193). Inset: standard boxplot depicting the measured proton permeability coefficient P for negatively charged (DOPC:DOPG, 3:1 w/w) electroformed (EF) (*yellow*, *n* = 29) and octanol-assisted liposome assembly (OLA) (*red*, *n* = 107) GUVs and uncharged DOPC OLA-GUVs (*blue*, *n* = 25). The white circles represent the mean. Both PC and PC:PG OLA-GUVs were prepared using the same buffer as in the main figure, but with the addition of 15 vol% glycerol, which was found to have no effect on proton permeability (see supporting material). (*C*) Calculation of transmembrane potential Δψ from a flux profile across a single GUV by solving the GHK equation (see main text) using numerical root finding. The resultant solution is indicated by the black dashed line. Inset: transmembrane potential buildup as extracted from the flux profile shown in the main figure. To see this figure in color, go online.

Fig. 2*A* shows the resultant flux profiles $\text{J}\left(\Delta \left[{\text{H}}^{+}\right]\right)$, analyzed from the intensity curves depicted in Fig. 1 *C*. As can be seen, the flux increase saturates as $\Delta \left[{\text{H}}^{+}\right]$ increases, indicating that a transmembrane (diffusion) potential Δψ is gradually accumulating across the GUV membrane (see below). Taking into account that the leakage rate of HCl, in its neutral form, is orders of magnitude higher than that of a proton (H^+^) (41) and that at neutral pH HCl is completely dissociated, our result implies that protons cross the membrane predominantly as H^+^ and not HCl, as previously suggested to account for their exceptionally high permeability (22,42). On the other hand, at very small values of $\Delta \left[{\text{H}}^{+}\right]$, i.e., when the change of lumenal pH is typically ${\text{dpH}}_{\text{i}}\left(\text{t}\right)<0.1$, the flux changes linearly (Fig. 2 *A*, *inset*), implying that Δψ is negligible. Hence, within this linear regime the permeability coefficient of protons can be directly extracted for each vesicle through ${\text{P}}_{{\text{H}}^{+}}={\text{J}}_{{\text{H}}^{+}}/\Delta \left[{\text{H}}^{+}\right]$.

Fig. 2*B* shows the distribution of ${\text{P}}_{{\text{H}}^{+}}$ values obtained from different single-vesicle permeation measurements following the addition of HCl. As can be seen, the obtained distribution plot is slightly skewed to the right as only positive ${\text{P}}_{{\text{H}}^{+}}$ values are possible. Nevertheless, the single peak at ${\text{P}}_{{\text{H}}^{+}}=0.002$ cm/s indicates a single population of vesicles with a similar membrane permeability to protons, implying that their translocation mechanism is not dictated by rare processes such as the formation of hydrated (transient) pores in the lipid bilayer, as previously suggested by Kuyper et al. (31). We note, however, that, unlike in the current study, Kuyper et al. examined the permeation of protons across small unilamellar vesicles, adsorbed to a glass surface, by rapidly changing the pH of the external solution from 9 to 3.5—conditions which may impose higher tension on the lipid bilayer and possibly promote pore formation.

The obtained permeability coefficients shown in Fig. 2 *B* agree with previously reported ${\text{P}}_{{\text{H}}^{+}}$ values (10^−4^–10^−3^ cm/s) (8,18,19,32,43,44). However, it was suggested by Cafiso and Hubbell (45) that ${\text{P}}_{{\text{H}}^{+}}$ is in fact much smaller, in the range of 10^−9^–10^−7^ cm/s, as also reported by others (21,22,31), and that larger permeability coefficients may obtain when trace organic contaminants reside in the lipid bilayer. Since OLA-GUVs may possibly contain residual octanol molecules in their membrane, we measured the permeability of protons across electroformed GUVs (Fig. S6), prepared using the same buffer and lipid composition, to examine whether octanol contributes to the large permeability coefficients obtained in our experiments. The inset to Fig. 2 *B* shows the obtained ${\text{P}}_{{\text{H}}^{+}}$ values following the addition of HCl to electroformed DOPC:DOPG (3:1 w/w) GUVs. As can be seen, the range of obtained values is comparable with that of OLA-GUVs, indicating that proton permeation is not affected by the presence of residual octanol in the membrane.

Another membrane property that may influence the leakage rate of protons in our measurements is the negative surface potential of GUVs (DOPC:DOPG, 3:1). To examine whether ${\text{P}}_{{\text{H}}^{+}}$ is affected by the surface potential of the lipid bilayer, we prepared uncharged OLA-GUVs that were composed only of DOPC (zwitterionic phospholipid) and measured their permeability to protons following the addition of HCl. Analysis of the obtained flux profiles revealed that the permeation rate of protons across DOPC GUVs is indeed lower than that of negatively charged GUVs prepared under the same experimental conditions (Fig. S7), with average permeability coefficients of ${\bar{\text{P}}}_{{\text{H}}^{+}}\left(\text{PC}\right)=8.3\times 10^{-4}$ cm/s and ${\bar{\text{P}}}_{{\text{H}}^{+}}\left(\text{PC}:\text{PG}\right)=1.9\times 10^{-3}$ cm/s, respectively (Fig. 2 *B*, *inset*). Furthermore, our finding that ${\bar{\text{P}}}_{{\text{H}}^{+}}\left(\text{PC}:\text{PG}\right)/{\bar{\text{P}}}_{{\text{H}}^{+}}\left(\text{PC}\right)\approx 2$ emphasizes the fact that while electrostatic interactions between ions and the lipid bilayer influences their flow across it, the solvation (Born) energy is the dominating energy barrier for ionic leakage across biological membranes (46).

### Quantification of electrochemical gradients across single vesicles

We elucidated the influence of Δψ on proton flux by calculating the flux profile of protons in the absence of Δψ using ${\text{P}}_{{\text{H}}^{+}}=3.5\times 10^{-3}$ cm/s as the average permeability coefficient of all flux profiles in the inset to Fig. 2 *A*. A comparison between the expected flux in the absence of electric potential (*black dashed lines*) and the measured flux profiles in Fig. 2 *A* suggests that, in our system, Δψ is significant enough to modulate the flux of protons at small concentration gradients $\Delta \left[{\text{H}}^{+}\right]∼0.2\times 10^{-10}$ mol/cm^3^ (= 20 *μ*M). The accumulation of transmembrane potential during proton permeation can be quantified by solving the GHK flux equation for any measured flux profile, using numerical root finding, with Δψ as the only free parameter (all other parameters are measured in the experiment). Fig. 2 *C* demonstrates the solution of the GHK flux model for a flux profile across a single GUV. The resultant diffusion potential (Fig. 2 *C*, *inset*) reveals the gradual buildup of voltage across the membrane as more protons pass through it. Subsequently, the corresponding electrochemical proton gradient, also referred to as the proton motive force (pmf), can be calculated by $\text{pmf}=\Delta \text{ψ}-2.3\frac{\text{RT}}{\text{F}}\left({\text{pH}}_{\text{i}}-{\text{pH}}_{\text{o}}\right)$ to assess the available pmf from passive leakage in our system (47). Likewise, our approach can be utilized to determine the necessary electrochemical gradients for activating membrane proteins, such as ATP synthase and bacterial efflux pumps, in artificial systems such as proteoliposomes.

**Figure 3:**
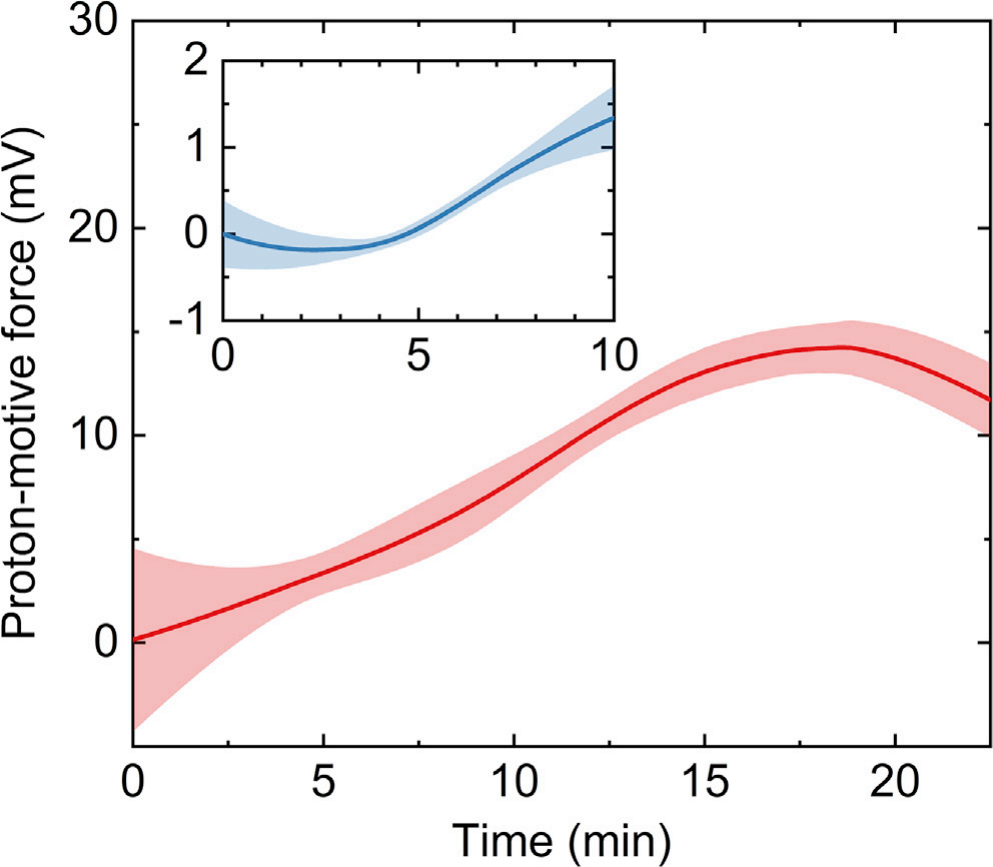
Development of the proton motive force (pmf) following the addition of HCl to DOPC:DOPG (3:1 w/w) GUVs (the relevant flux profile is shown in Fig. 2*A*). Inset: development of the pmf following the addition of formic acid (Fig. 4*A*). The *solid lines* and *bands* represent the average and standard deviation of the measured pmf, respectively. To see this figure in color, go online.

Fig. 3 shows the evolution of the pmf for the proton flux measurements shown in Fig. 2, with a maximum average value of pmf=14.2 mV (at T=21°C) for the average values: Δψ=45 mV and ΔpH≈0.53. The relatively low measured pmf values, compared with bioenergetic membranes (48,49), and appearance of a maximum point in the pmf curve emanate from the presence of two opposing driving forces in our measurements, where ΔpH acts to translocate protons into the vesicle while Δψ acts to move them outside. As such, our measurements suggest that alteration of proton concentration in the external solution may not be sufficient to generate biologically relevant pmf values (≈130–200 mV (48, 49)). Still, in principle, higher pmf values can be achieved through ensuring that both driving forces act in the same direction, for instance, by inducing a negative Δψ with efflux of ions such as K^+^, using ionophores like valinomycin, and then acidifying the external solution (7).

### Effect of conjugated anion on electrochemical potential generation

**Figure 4:**
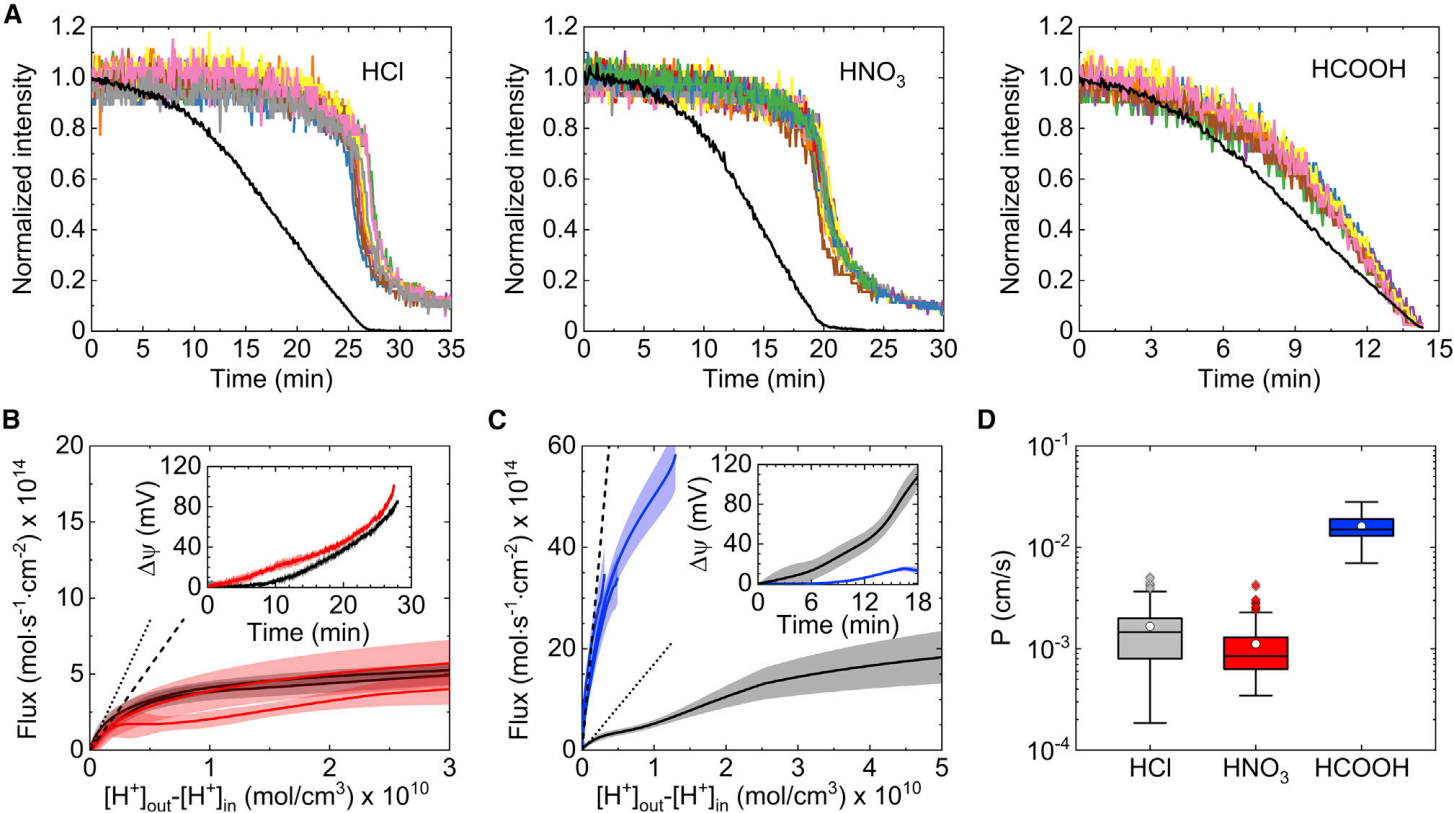
Anion-dependent permeability of protons. (*A*) Time-resolved plots of extravesicular (*black line*) and lumenal (*colored lines)* pyranine intensity following the addition of HCl, HNO_3_, and formic acid (HCOOH) to DOPC/DOPG (3:1 w/w) GUVs. (*B*) Flux profiles of protons measured for HNO_3_ (*red curves*) and HCl (*black curves*). The bold lines and bands represent the average flux profile and standard deviation of all profiles taken for a set of vesicles, respectively. The black dashed and dotted lines are the concatenate fit of the flux equation ${\text{J}}_{{\text{H}}^{+}}=\text{P}\Delta \left[{\text{H}}^{+}\right]$ to the linear regime (not shown) of the plotted HNO_3_ and HCl flux profiles, respectively. The comparison between the two acids was repeated twice using different samples of vesicles. Inset: corresponding transmembrane potential buildup for HCl (*black*) and HNO_3_ (*red*). (*C*) Flux profiles of protons measured for formic acid (*blue curves*) and HCl (*black curves*). The black dashed and dotted lines are the concatenate fit of the flux equation to the linear regime (not shown) of the plotted HCOOH and HCl flux profiles, respectively. Inset: corresponding transmembrane potential buildup for HCl (*black*) and formic acid (*blue*). (*D*) Standard boxplot of proton permeability coefficients as measured from the flux profiles of HCl (*n* = 131), HNO_3_ (*n* = 133), and HCOOH (*n* = 21). The white circles represent the mean *p*-value. To see this figure in color, go online.

Weak organic acids are reported to leak through the lipid bilayer at higher rates than strong inorganic acids, suggesting a distinct translocation route for each type of acid. To elucidate whether electrochemical gradients can be generated using weak acids, we measured the passive proton permeation of formic acid (HCOOH) and compared it with that of two strong acids, HCl and HNO_3_. As can already be seen from the intensity plots in Fig. 4 *A*, while the permeation rate of protons appears to be comparable for HCl and HNO_3_ it is significantly higher when formic acid is added. The observed difference between weak and strong acids becomes even more evident by analyzing their resultant flux profiles $\text{J}\left(\Delta \left[{\text{H}}^{+}\right]\right)$.

Fig. 4*B* depicts the flux profiles of protons following the addition of HCl (*black curves*) and HNO_3_ (*red curves*). The fact that the HNO_3_ profiles saturates at high $\Delta \left[{\text{H}}^{+}\right]$ indicates that, as in the case of HCl, there is a significant buildup of transmembrane potential, since protons mainly cross the membrane as H^+^. This strong deviation from linearity is manifested by the clear difference between $\text{J}\left(\Delta \left[{\text{H}}^{+}\right]\right)$ and the calculated proton flux for HNO_3_ (Fig. 4 *B*, *black dashed line*) when Δψ=0 mV and the corresponding measured permeability coefficient is ${\text{P}}_{{\text{H}}^{+}}=\left(1.1\pm 0.8\right)\times 10^{-3}$ cm/s (Fig. 4 *D*). For comparison, the black dotted line in Fig. 4 *B* illustrates the equivalent permeation rate of protons when HCl is added, where ${\text{P}}_{{\text{H}}^{+}}=\left(1.7\pm 1.2\right)\times 10^{-3}$ cm/s (Fig. 4 *D*). Furthermore, the overlapping flux profiles and diffusion potentials (Fig. 4 *B*, *inset*) of both acids imply that Cl^−^ and NO_3_^−^ translocate through the membrane at similar rates, as previously mentioned by others (50).

On the contrary, we found that in the case of formic acid, the measured flux varies almost linearly with concentration gradient (Fig. 4 *C*), indicating that the accumulation of charge in the vesicle’s interior is significantly lower relative to HCl and HNO_3_, as further demonstrated by the difference between the obtained Δψ values for each acid (Fig. 4 *C*, *inset*). Consequently, a very low maximal average electrochemical gradient of pmf=1.3 mV (at T=21°C) was generated across the vesicle membrane for the average values: Δψ=4.7 mV, ΔpH≈0.05 (Fig. 3, *inset*). The near linear nature of the formic acid flux profile and its variation from the obtained HCl profiles, obtained using the same vesicle sample, are emphasized by the respective dashed and dotted black lines in Fig. 4 *C*. Altogether, permeation analysis provides clear evidence that in the case of formic acid, protons translocate through the lipid bilayer mainly in the form of an uncharged acid (HCOOH) and then dissociate in the vesicle lumen. Accordingly, the permeability coefficient for formic acid ${\text{P}}_{{\text{H}}^{+}}=\left(1.6\pm 0.6\right)\times 10^{-2}$ cm/s was found to be an order of magnitude larger than ${\text{P}}_{{\text{H}}^{+}}$ measured for HCl and HNO_3_ (Fig. 4 *D*), in agreement with previously reported values (30,51). Similarly, a minimum point appears in the pmf curve of formic acid (Fig. 3, *inset*) due to the rapid development of ΔpH compared with Δψ, which rises slowly owing to permeation of undissociated acid molecules.

**Figure 5:**
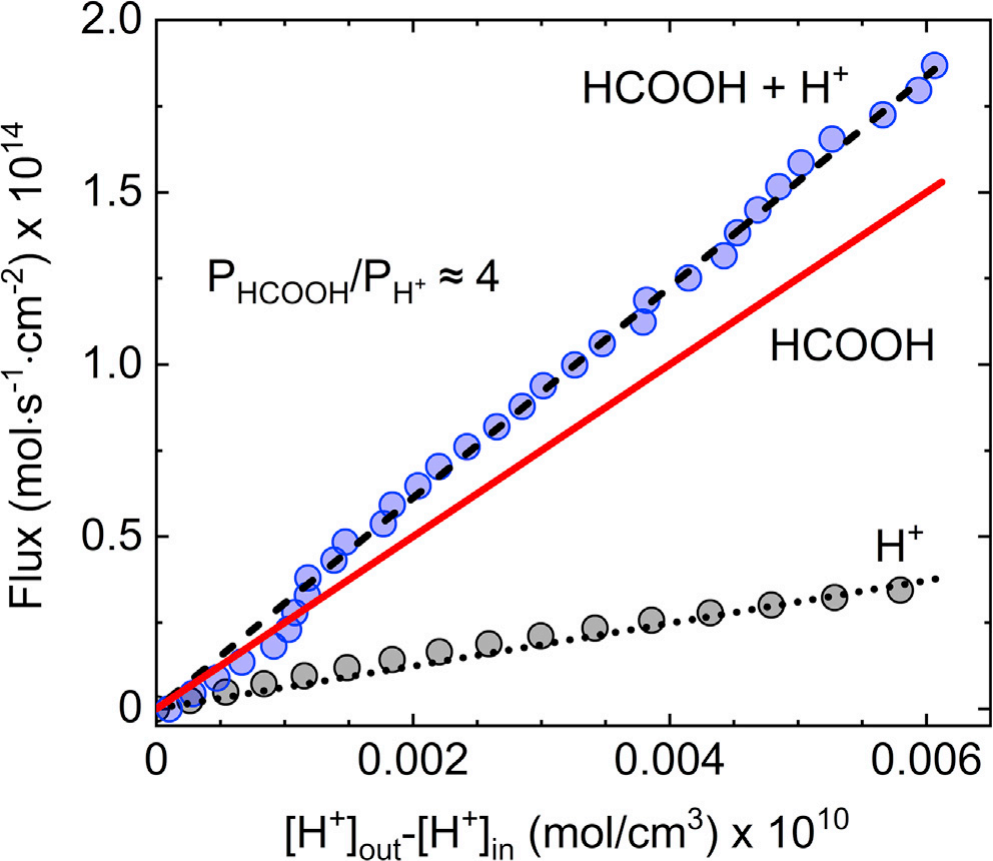
Elucidation of formic acid permeation pathway. The gray and blue circles represent the average flux profiles of protons across DOPC/DOPG (3:1 w/w) GUVs following the addition of HCl and formic acid, respectively. The black dashed and dotted lines are the corresponding least-squares fits of ${\text{J}}_{{\text{H}}^{+}}=\text{P}\Delta \left[{\text{H}}^{+}\right]$ to the data with ${\bar{\text{P}}}_{{\text{H}}^{+}}\left(\text{HCOOH }+{\text{ H}}^{+}\right)=3.1\times 10^{-2}\pm 1.8\times 10^{-4}$ cm/s and ${\bar{\text{P}}}_{{\text{H}}^{+}}\left(\text{HCl}\right)=6.2\times 10^{-3}\pm 1.2\times 10^{-4}$ cm/s. The red solid line is the flux profile of the undissociated formic acid, estimated through subtracting the black dotted line (flux of H^+^) from the black dashed line (flux of HCOOH + H^+^). The slope of the red curve is the evaluated permeability coefficient of formic acid, ${\bar{\text{P}}}_{\text{HCOOH}}=2.5\times 10^{-2}\pm 2.2\times 10^{-4}$ cm/s. To see this figure in color, go online.

To verify that proton leakage is indeed governed by a flux of undissociated acid, we quantified the relative portion of permeated HCOOH and H^+^ by comparing between the averaged flux profiles of HCl and formic acid at small $\Delta \left[{\text{H}}^{+}\right]$ values, namely, at the linear regime when Δψ≈0 mV for both acids (Fig. 5). Under the reasonable assumption that only dissociated protons (H^+^) cross the membrane when HCl is added (Fig. 2 *A*) and that the permeability rate of the weak acid anion (HCOO^−^) is a few orders of magnitude smaller than that of HCOOH (17, 28, 29) thus also of H^+^, the flux of the undissociated acid (Fig. 5, *red solid line*) can be estimated by ${\text{J}}_{\text{HCOOH}}={\text{J}}_{\text{FA}}-{\text{J}}_{{\text{H}}^{+}},$ where ${\text{J}}_{\text{FA}}$ and ${\text{J}}_{{\text{H}}^{+}}$ are the flux profiles of formic acid (i.e., of both HCOOH and H^+^) and HCl, respectively. The relative permeability of HCOOH and H^+^ (i.e., the ratio between the slopes of the red and black dotted lines) can then be obtained through $\frac{{\text{J}}_{\text{HCOOH}}}{{\text{J}}_{{\text{H}}^{+}}}=\frac{{\text{P}}_{\text{HCOOH}}}{{\text{P}}_{{\text{H}}^{+}}}$. Here we assume that the flux of HCOOH is governed by Δ[H^+^] since at pH 7.6 the concentration of the undissociated acid is negligible compared with H^+^ according to $\frac{\left[{\text{A}}^{-}\right]}{\left[\text{HA}\right]}=10^{\text{pH}-{\text{pK}}_{\text{a}}}\approx 10^{4}$. We found that within our experimental conditions ${\text{P}}_{\text{HCOOH}}/{\text{P}}_{{\text{H}}^{+}}=4.0\pm 0.1$, meaning that 20% of the protons that traverse into the vesicles lumen cross the membrane as H^+^ while the rest permeate as HCOOH. Therefore, while our results show that the governing translocation pathway of weak acids such as HCOOH is via simple diffusion of the undissociated acid, as reported earlier (28, 29, 30), the leakage of proton ions and the corresponding accumulation of transmembrane potential cannot be ignored. In addition, since the portion of permeated protons is expected to increase with increasing hydrophilicity of the protonated acid (28,52), our finding indicates that weak acids can be used to generate electrochemical gradients in artificial cell systems.

## Conclusions

To conclude, we directly quantified the permeation coefficient and flux of protons across single cell-sized lipid vesicles under modulated electrochemical gradients. Through analyzing the obtained flux profiles, we determined the accumulation of transmembrane potential (i.e., level of membrane polarization) as a result of proton permeation following the addition of different types of acids. Our results reveal that in the case of a weak acid (formic acid), protons accumulate in the vesicle interior owing to the synchronous permeation of uncharged acid molecules (AH) and protons (H^+^), while in the case of strong acids (HCl or HNO_3_) proton permeation was governed by translocation of H^+^. Accordingly, a larger pmf was obtained for strong acids (pmf=14.2 mV) relative to formic acid (pmf=1.3 mV). Further analysis of proton flux, following the addition of formic acid, revealed that out of the total amount of permeated protons, a fraction of ≈0.2 traverses the membrane as H^+^, with the rest (≈0.8) in the form of a neutral acid. As such we show that, using our approach, the correlation between proton permeation and pmf can be elucidated for different types of acids. We anticipate that this approach can be employed for resolving protein-based transport of protons using biomimetic giant vesicles and molecular dynamic simulations and will guide future efforts to unravel the membrane permeation of vital acids, including fatty acids and carboxylic acid-containing drugs.

## Author contributions

R.T. designed the experiments, conducted the measurements, analyzed the data, and wrote the manuscript. M.F. assisted with coding. M.F. and U.F.K. assisted with theoretical aspects of ion transport. All authors discussed the results and commented on the manuscript.

## Supporting material

Document S1. Figures S1–S7

Document S2. Article plus supporting material
