# Supplementary material for: Quantifying proton-induced membrane polarization in single biomimetic giant vesicles
Source: Biophys J. 2022 May 28;121(12):2223–32. doi: 10.1016/j.bpj.2022.05.041 (PMC9279353; doi:10.1016/j.bpj.2022.05.041)
Supplement: Document S1. Figures S1–S7 [file mmc1.pdf]

Biophysical Journal, Volume *121*

Supplemental information

Quantifying proton-induced membrane polarization in single biomimetic giant vesicles

Ran Tivony, Marcus Fletcher, and Ulrich F. Keyser

Supplementary information for:

Quantifying proton-induced membrane polarization in single biomimetic giant vesicles

Ran Tivony*, Marcus Fletcher and Ulrich F Keyser

Cavendish Laboratory, University of Cambridge, JJ Thomson Avenue, Cambridge CB3 0HE, UK.

* Corresponding author: E-mail: rt497@cam.ac.uk

Table of content

S1. Photobleaching of pyranine 2

S2. Verifying the absence of pyranine leakage during permeation measurements 2

S3. pH-dependent variation of pyranine intensity 3

S4. The effect of added acid amount on dissipation rate of proton gradients 4

S5. Buffer capacity 5

S6. Proton permeation across negatively charged electroformed GUVs (DOPC:DOPG) 6

S7. The effect of lipid-bilayer charge on proton permeability 6

S1. Photobleaching of pyranine

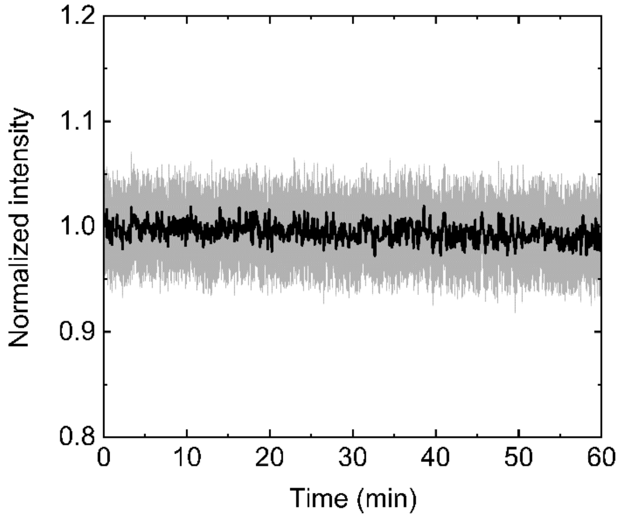

Figure S1. Photobleaching of pyranine. The normalized intensity of pyranine, encapsulated within the GUVs lumen, was measured through a confocal microscope using the same acquisition rate (0.2 frames/sec) and laser power as in proton permeation experiments shown in the main paper. The black line and gray band are the average normalized intensity and standard deviation of all profiles taken for a set of vesicles (n = 89).

S2. Verifying the absence of pyranine leakage during permeation measurements

To accurately quantify the inward flux of protons we verified that the measured reduction of pyranine intensity is due to proton permeation and not as a result of pyranine leakage out of the vesicles. For this purpose, we first added HCl to initiate the leakage of protons inside the GUVs and then (after ~10min) we increased the pH of the external solution by adding 1µl of 1M NaOH. Therefore, in case that the observed drop in fluorescence signal, following acidification with HCl, is indeed due to pyranine leakage out of the vesicles, no change in pyranine intensity is expected to occur upon raising the pH value of the GUVs interior. Figure S4A shows the gradual decrease of pyranine intensity as a result of decreasing the pH outside of the vesicles, where the black trace is the extravesicular solution and each coloured trace signifies the lumen of a single GUV. As can be seen in figure S4B, upon adding NaOH, the

fluorescence intensity inside the vesicles (coloured traces) progressively increases towards the intensity of the of the external solution (black trace), indicating that pyranine remained encapsulated in the GUVs during proton permeation.

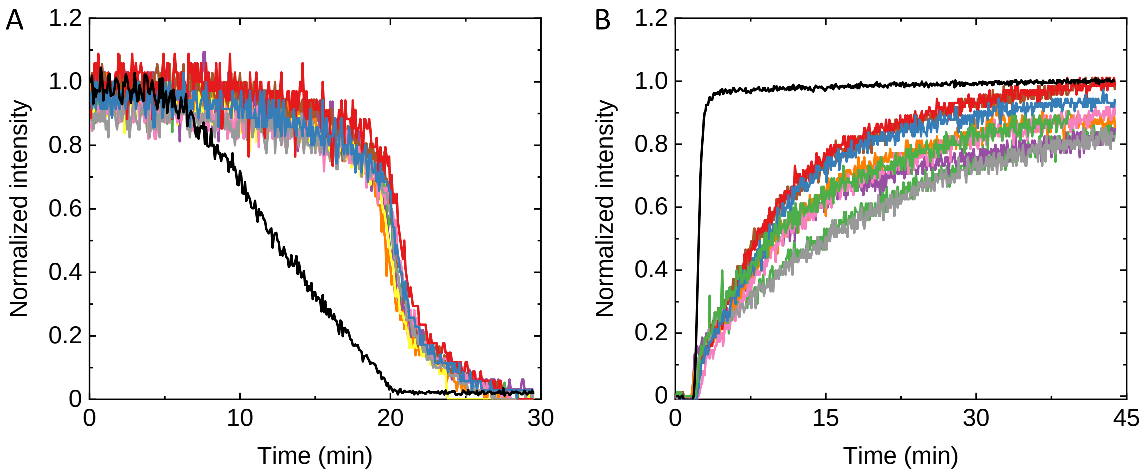

Figure S2. Pyranine fluorescence intensity inside GUVs following acidification (A) and basification (B) of the external solution. The black and coloured traces signify the intensity of the extravesicular solution and GUVs lumen, where each trace in A and B is colour-coded to match the same GUV.

S3. pH-dependent variation of pyranine intensity

To quantify the pH from intensity data, we measured the intensity of pyranine (HPTS) at different pH values and under the same conditions in which the permeation experiments were conducted (figure S1). Fluorescence measurements were taken by a confocal microscope and pyranine was dissolved in buffer A' (see main text) to a final concentration of 10µM. Consequently, the pH value the pH inside and outside every detected GUVs was calculated from the obtained pyranine intensity (I) using, $\mathrm{pH} = 0.53\ln(\mathrm{I}) + 7.65$.

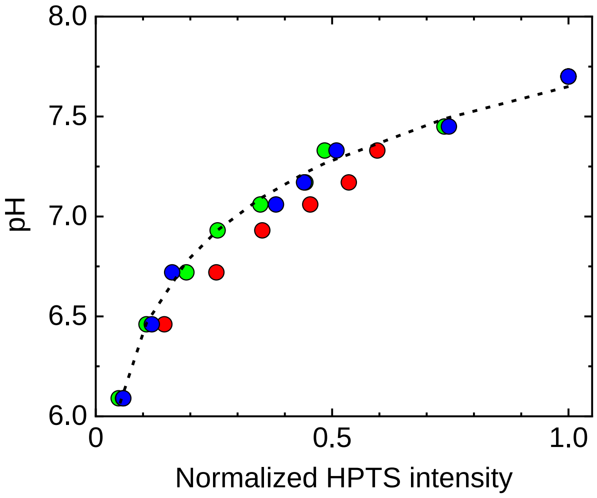

Figure S3. pH-dependent intensity of pyranine. Each symbol represent a separate measurement and the black dashed line represents the best fit to data using least squares regression.

S4. <u>The effect of added acid amount on dissipation rate of proton gradients</u>

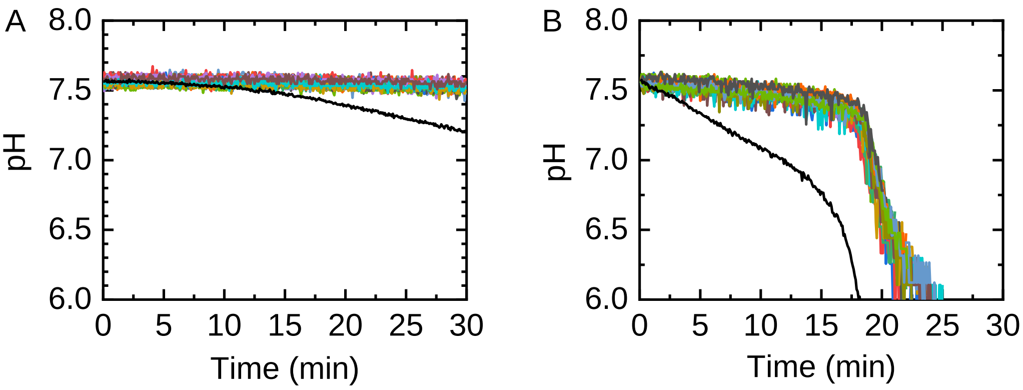

Figure S4. Quantification of lumenal (coloured lines) and extravesicular (black lines) pH values following the addition of 1µl (A) and 1.5µl (B) 1% HCl to the GUVs solution (60µl). The corresponding acid concentrations in the GUVs solution are 5.4mM and 8mM, respectively.

S5. Buffer capacity

The buffer capacity, β, indicates the efficiency of a buffer to resist its change of pH following an addition of n moles of protons ($\beta = dn/dpH$). Since buffer capacity depends on the pH value of the buffer, we measured $\beta(pH)$ by titrating the buffers used in this study (see experimental section) with known amounts of protons (dn), and analyzed the resultant pH change (dpH) at various pH values. The obtained $\beta(pH)$ curves were fitted using a polynomial equation to calculate the buffer capacity at any measured lumenal pH. The concentration of protons in the GUVs interior was then extracted using the following relation $[H^+]_i = \beta(pH) \times dpH/V$, where V is the vesicles volume.

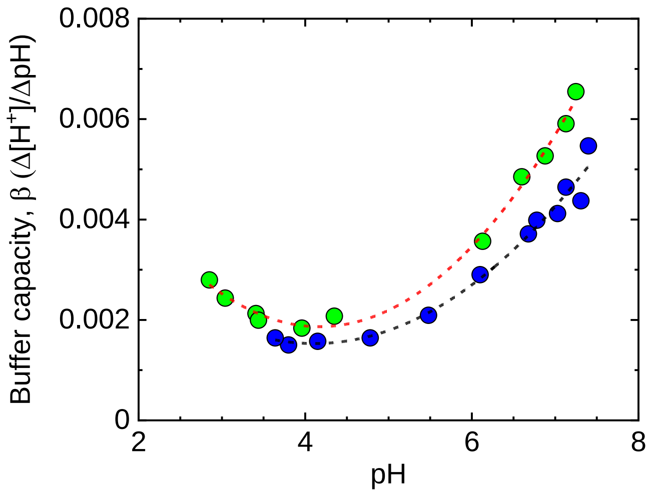

Figure S5. The blue and green circles are the $\beta(pH)$ values measured for buffer A' with and without 15% v/v glycerol. The and red and black dashed line are the best fit of a polynomial to the data using least squares. The following equations were obtained for the red and black fits: $\beta = 0.0005 \times pH^2 - 0.004 \times pH + 0.0102$ and $\beta = 0.0003 \times pH^2 - 0.0027 \times pH + 0.007$, respectively.

S6. <u>Proton permeation across negatively charged electroformed GUVs (DOPC:DOPG)</u>

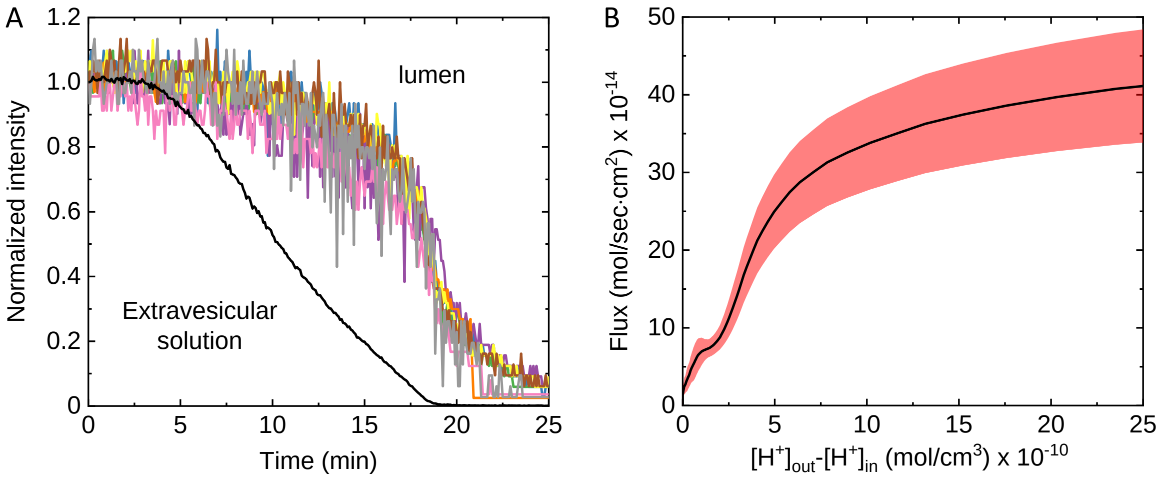

Figure S6. Proton permeation measurement across electroformed GUVs (DOPC:DOPG, 3:1 w/w) under the same conditions and buffer used for proton permeation measurement using OLA GUVs (see experimental data). **A.** Time-resolved fluorescence intensity of pyranine following the addition of HCl. The black curve indicates the intensity change outside the vesicles and the coloured curves indicates the intensity change of the lumen, where each curve represents a single GUV. **B.** Flux profiles of protons measured across the GUVs depicted in figure S6A. The bold black line and red band represent the average flux profile and standard deviation of all profiles taken for a set of vesicles, respectively.

S7. <u>The effect of lipid-bilayer charge on proton permeability</u>

To examine the effect of surface charge on the leakage rate of protons P_{H+}, we prepared uncharged GUVs from the zwitterionic phospholipid DOPC (1,2-dioleoyl-sn-glycero-3-phosphocholine) and measured their permeability to protons following the addition of HCl. Since DOPC GUVs were found to be less stable than negatively charged DOPC:DOPG GUVs in the buffer solution used in our experiments (see experimental section), we used the same buffer but with glycerol (buffer A + 15% v/v glycerol). To confirm that glycerol does not affect the permeability of the lipid bilayer, we prepared DOPC:DOPG GUVs in 15% glycerol buffer and compared the obtained flux profile and permeability coefficient of protons (figure S5A,

red curves) to that of DOPC:DOPG GUVs that were prepare in the same buffer but without glycerol (figure S5B).

As can be seen, similar flux profiles and permeability coefficients (inset to figure S5B) were obtained in the presence and absence of glycerol, indicating that, at this concentration, glycerol does not influence the passive leakage of protons through the vesicles membrane. On the other hand, the flux of protons across DOPC GUVs (figure S5A, blue curves) was found to be lower than that of negatively charged vesicles. In addition, lower permeability coefficients were measured for the uncharged vesicles ($\overline{P}_{H^+}(PC) = 8.3 \times 10^{-4}$ cm/s vs. $\overline{P}_{H^+}(PC:PG) = 1.9 \times 10^{-3}$ cm/s) as demonstrated by the linear flux profiles shown in the inset to figure S5A. Altogether, our results suggest that the membrane permeability to protons (and most likely for other ions) is affected, though not dramatically, by the surface charge of the lipid vesicles.

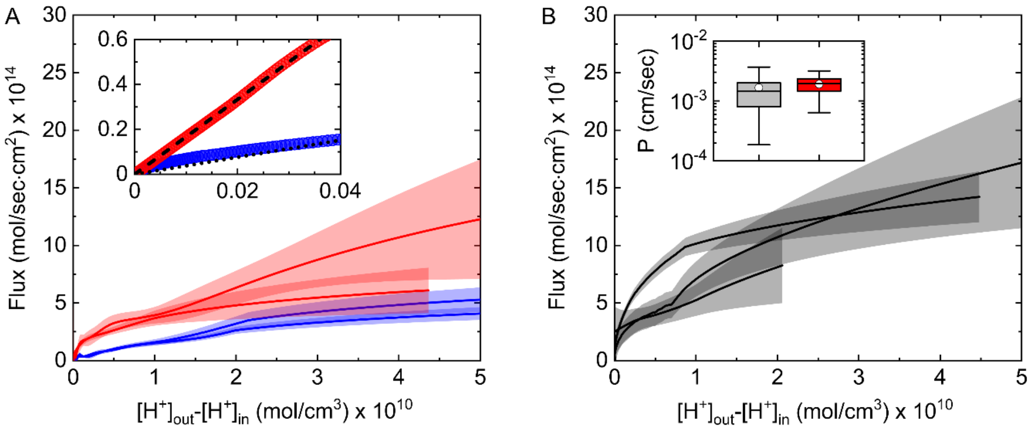

Figure S7. Influence of membrane charge on proton permeability. **A.** Flux profiles of protons measured in 15% glycerol buffer for DOPC:DOPG negatively charge GUVs (red curves) and DOPC uncharged GUVs (blue curves) following the addition of HCl. The bold lines and bands represent the average flux profile and standard deviation of all profiles taken for a set of vesicles. The comparison between the two types of vesicles was repeated twice using different samples of vesicles. Inset: average flux profiles of protons at small concentration gradients colour-coded to match the flux curves in the main figure. **B.** Flux profiles of protons measured in buffer A (no glycerol) for DOPC:DOPG negatively charge GUVs following the addition of HCl. Inset: standard box plot of proton permeability coefficients in the absence (gray) and presence (red) of 15% v/v glycerol.
