# Supplementary material for: Quantifying proton-induced membrane polarization in single biomimetic giant vesicles
Source: Biophys J. 2022 May 28;121(12):2223–32. doi: 10.1016/j.bpj.2022.05.041 (PMC9279353; doi:10.1016/j.bpj.2022.05.041)
Supplement: Document S2. Article plus supporting material [file mmc2.pdf]

Biophysical Journal
Article

Biophysical Society

Quantifying proton-induced membrane polarization in single biomimetic giant vesicles

Ran Tivony,[1,*] Marcus Fletcher,[1] and Ulrich F. Keyser[1]
[1]Cavendish Laboratory, University of Cambridge, Cambridge, United Kingdom

ABSTRACT Proton gradients are utilized by cells to power the transport activity of many membrane proteins. Synthetic cells, such as proteo-giant unilamellar vesicles, offer an advanced approach for studying the functionality of membrane proteins in isolation. However, understanding of protein-based transport in vitro requires accurate measurements of proton flux and its accompanying electrochemical gradient across the lipid bilayer. We present an approach to directly quantify the flux of protons across single cell-sized lipid vesicles under modulated electrochemical gradients. Our measurements reveal the corresponding association between proton permeation and transmembrane potential development and its relation to the chemical nature of the conjugated anion (base). In the case of formic acid, we showed that, out of the total amount of permeated protons, a fraction of $\approx$0.2 traverse the lipid bilayer as H^+, with the rest ($\approx$0.8) in the form of a neutral acid. For strong acids (HCl or HNO_3), proton permeation was governed by translocation of H^+. Accordingly, a larger proton motive force (pmf) was generated for strong acids (pmf $= 14.2$ mV) relative to formic acid (pmf $= 1.3$ mV). We anticipate that our approach will guide the development of protein-based transport driven by proton gradient in artificial cell models and enable a deeper understanding of how vital acids, such as fatty acids, amino acids, bile acids, and carboxylic acid-containing drugs, traverse the lipid bilayer.

SIGNIFICANCE Cells generate and harness electrochemical proton gradients to drive the transport activity of various membrane proteins, from ATP synthase to bacterial efflux pumps. To elucidate the functionality of these proteins in artificial cell models, it is essential to resolve the correlation between electrochemical gradient evolution and proton permeation across the lipid bilayer. For this purpose, we directly quantified proton flux under modulated electrochemical gradients across single cell-sized lipid vesicles. We were able to differentiate between protons that translocate as ions or in the form of an uncharged acid and determine the corresponding development of transmembrane potential. Our results indicate a suitable approach for elucidating protein-based transport in synthetic cell systems.

INTRODUCTION

Ion flux across biological membranes is vital for regulating cellular processes and maintaining homeostasis (1). In cells and organelles, ions are translocated primarily through channels and pumps (2) but can also passively leak through the phospholipid bilayer (3). In fact, these two permeation routes are coupled and operate synchronously to modulate the electrochemical gradients that propel many essential cellular activities such as transport of nutrients, organelle acidification, and transmission of electrical signals (4). Therefore, accurate quantification of ionic movement and corresponding electrochemical gradients across the lipid bilayer is important for discriminating protein-mediated transport from passive permeation in transport studies using artificial cell systems (5–9).

Synthetic lipid vesicles (liposomes), spherical compartments enclosed by a phospholipid bilayer, have proven a successful biomimetic system for scrutinizing the movement of ions through biological membranes (10–12). Over the last decades, vesicle-based transport studies have provided a wealth of information about the passive leakage of ions and their permeability coefficient (P) (13–18), a fundamental property that quantifies the rate at which a solute crosses the membrane. A prominent example is the finding that protons can pass through the lipid bilayer with exceptionally high rates ($P_{H^+} \approx 10^{-4}$ cm/s), several orders of magnitude larger than other biologically relevant monovalent ions such as Na^+ and K^+ (19,20). To account for their anomalously high permeability, Paula et al. showed that proton leakage involves their diffusion (i.e., hopping) through hydrated defects in the membrane (i.e., the transient pore

Submitted March 20, 2022, and accepted for publication May 24, 2022.
*Correspondence: rt497@cam.ac.uk
Editor Name: Heiko Heerklotz.
https://doi.org/10.1016/j.bpj.2022.05.041
© 2022 Biophysical Society.
This is an open access article under the CC BY license (http://creativecommons.org/licenses/by/4.0/).

mechanism) rather than by a simple diffusion across the nonpolar hydrocarbons of the lipid bilayer (i.e., the solubility-diffusion mechanism) (16). Nonetheless, since the obtained proton permeation coefficients (P_{H^+}) were found to differ by several orders of magnitude between various studies (18,19,21–24), the origin of proton leakage is still under intense scrutiny (25,26). One source for discrepancies in P_{H^+} arises from the fact that the majority of ion permeation studies to date were performed using an ensemble of vesicles, producing averaged permeability coefficients that only reflect the collective property of the examined dispersion.

Fundamentally, protons are utilized by cells to drive the biochemical activity of various membrane proteins, from ATP synthase to bacterial efflux pumps, through generation of electrochemical gradients (or a proton motive force [pmf]). Artificial cell models, such as proteoliposomes, offer an advanced approach to study membrane proteins in isolation (27). However, interpretation of protein functionality in synthetic cells relies on accurate determination of proton fluxes and resultant electrochemical gradients (8). For instance, it is generally accepted that weak organic acids diffuse through the lipid bilayer in their neutral form (HA) following protonation of the conjugated anion (A^-) close to the membrane surface (28–30). In the case of strong inorganic acids, however, it is believed that only dissociated protons (H^+) cross the membrane (8,19,31), indicating that the generation of electrochemical gradients due to proton permeation varies with the chemical nature of their conjugated anion. As such, quantifying the fraction of permeated charged and uncharged species (i.e., HA and H^+), for a given acid, is essential for resolving the level of membrane polarization and, subsequently, the correlation between proton motive force (pmf) and protein activity in vitro.

Unlike bulk permeability assays, in which the properties of the liposome population vary widely and cannot be precisely measured (12), single-vesicle analysis provides information at a higher level of detail, enabling the determination of ionic flux and permeability coefficient for each vesicle separately (8,31,32). Giant unilamellar vesicles (GUVs), cell-sized lipid vesicles, provide an even higher degree of precision, as they can be easily visualized under a light microscope while settled at the bottom of an observation chamber or trapped in a microfluidic chip (33). Likewise, their biologically relevant size makes GUVs an appropriate biomimetic model for studying transport phenomena in cells and organelles (5,6,9,34–36). Nevertheless, despite being extensively used as an established synthetic model for investigating membrane phenomena, there have been only limited attempts to employ GUVs for studying passive ion permeation (34–36).

Here, we describe an approach to resolve the correlation between the time-dependent flux and electrochemical gradients of protons across the lipid bilayer of cell-sized vesicles. Through this approach, we were able to determine the flux at different proton gradients and quantify the permeability coefficient and corresponding development of transmembrane potential (i.e., membrane polarization). By analyzing the gradient-dependent flux of protons, we showed that in the case of strong acids, such as HCl or HNO_3, proton permeation is governed by translocation of proton ions (H^+) under our experimental conditions. However, in the case of a weak organic acid such as formic acid, protons flow through the lipid bilayer both in the form of an undissociated acid (HA) and as H^+, generating lower diffusion potentials. We further evaluated the relative flux of each permeated form and found that, in the case of formic acid, only a small fraction (≈ 0.2) leaks as proton ions while the rest (≈ 0.8) leaks as a neutral acid. Consequently, we evaluated the maximal pmf generated for each type of acid.

MATERIALS AND METHODS

Materials

1,2-Dioleoyl-*sn*-glycero-3-phosphocholine (DOPC), 1,2-dioleoyl-*sn*-glycero-3-phospho-(1′-rac-glycerol) (sodium salt) (DOPG), 1,2-dioleoyl-*sn*-glycero-3-phosphoethanolamine-*N*-(lissamine rhodamine B sulfonyl) (ammonium salt) (18:1 Liss Rhod PE), and 1,2-dioleoyl-*sn*-glycero-3-phosphoethanolamine-*N*-(7-nitro-2-1,3-benzoxadiazol-4-yl) (ammonium salt) (18:1 NBD PE) were purchased from Avanti Polar Lipids (Alabaster, AL) as powder and dissolved in chloroform to a final concentration of 100 mg/mL (DOPC and DOPG) and 1 mg/mL (Liss Rhod PE and NBD PE). 8-Hydroxypyrene-1,3,6-trisulfonic acid trisodium salt (HPTS) was purchased from Merck (Kenilworth, NJ) and used as received. 1-Octanol was purchased from Sigma (St. Louis, MO) and used as received. Polydimethylsiloxane (PDMS) Sylgard 184 was purchased from Dow Corning (Midland, MI) and used as received.

Fabrication of microfluidic device

The PDMS microfluidic devices were fabricated using photolithography and soft lithography, as previously described elsewhere (37). The master mold for the octanol-assisted liposome assembly (OLA) design was prepared by spin-coating a thin layer of SU-8 2025 photoresist (Chestech, Rugby, UK) on a 4-inch silicon wafer (University Wafer, South Boston, MA), to generate a silicon master with feature heights around 20 μm. The wafer was then soft-baked (65°C for 1 min and at 95°C for 6 min), and the structures (designed in AutoCAD) were imprinted on the substrate with UV light using a tabletop laser direct imaging system (LPKF ProtoLaser; LPKF, Garbsen, Germany). The formed structures were then postbaked (1 min at 65°C and for 6 min at 95°C) and developed with propylene glycol monomethyl ether acetate. Finally, the wafer was hard-baked for 10 min at 120°C.

The PDMS chips were prepared by casting a degassed liquid PDMS (9:1 ratio with a curing agent) into the mold and then curing it for at least 2 h at 60°C. The hardened PDMS was taken off the mold and bonded to a PDMS-coated coverslip after oxygen plasma treatment for 10 s (100 W plasma power, 25 sccm, plasma etcher; Diener Electric, Ebhausen, Germany).

Preparation of giant unilamellar vesicles

GUVs were prepared using a microfluidic-based technique, OLA (38), which enables high-throughput and controlled production of monodispersed GUVs in various buffers and an excellent encapsulation efficiency. In brief, OLA uses flow focusing in a six-way junction to form GUVs

through dewetting of double-emulsion (w/o/w) capsules composed of an inner aqueous phase (IA), a lipid-octanol oil phase (LO), and an outer aqueous phase (OA). Operation of the microfluidic device is achieved via a pressure-driven pump by which flow rates of all three phases (IA, LO, and OA) can be tuned and monitored in real time. For all experiments, the base solution used for the IA and OA phases (prepared in Milli-Q water) consisted of buffer A: 10 mM HEPES and 200 mM sucrose, titrated with 1 M NaOH to reach a final pH value of 7.6. The IA (buffer A) also contained the membrane-impermeable pH-sensitive dye HPTS (pyranine, 10 μM), and the OA phase (pH 7.6) also included 50 mg/mL Kolliphor P-188 (Sigma-Aldrich, Gillingham, UK). The LO phase comprised 4 μL of a lipid stock solution (100 mg/mL DOPC:DOPG in ethanol; 3:1 v/v ratio), 1 μL of a fluorescent-lipid solution (1 mg/mL Liss Rhod PE in chloroform), and 95 μL of 1-octanol.

Electroformed GUVs were prepared using a Nanion Vesicle Prep Pro setup (Nanion, Munich, Germany). DOPC, DOPG, and Liss Rhod PE were dissolved in chloroform in a ratio of 3:1:0.01 w/w. The lipid mixture at 5 mg/ml (80 μl) was spin-coated on the conducting surface of an indium tin oxide (ITO)-coated glass slide (Nanion/VisionTek, Chester, UK). The chloroform was evaporated for 3 h in a desiccator, then 700 μL of buffer A (see above) with 10 μM HPTS was deposited within an O-ring chamber, which was then sealed with another ITO-coated slide. The electroformation protocol was carried out at 37°C and proceeded in three steps: 1) the AC voltage increased linearly from 0 to 3 V (peak to peak [p-p]) at 10 Hz over 40 min; 2) the voltage stayed at 3 V (p-p) and 10 Hz for 60 min; 3) the voltage decreased linearly to 0 V at 10 Hz over 20 min.

Data acquisition and visualization of proton permeation

Images and videos of GUVs were acquired by an inverted laser scanning confocal microscope (Olympus Fluoview FV1200, IX83; Olympus, Tokyo, Japan), using a 40× oil immersion objective (Olympus UPlanFL N). The lumen (HPTS labeled) and membrane (Liss Rhod-PE labeled) of the vesicles were visualized following their excitation with a 488 nm argon laser and 559 nm LED laser, respectively (the vesicle membrane was imaged for image analysis purposes—see below). Prior to their imaging, the GUVs were settled on a PDMS-coated glass slide (0.13 mm) in a custom-designed PDMS observation chamber by adding 20 μL of vesicles dispersion (without pyranine in the external solution) to 40 μL of buffer A′, an isosmotic glucose solution containing 10 mM HEPES, 200 mM glucose, and 15 μM pyranine (pH 7.6). The final pyranine concentration in the external solution was 10 μM.

Permeation experiments were performed after GUVs were left to sediment in the observation chamber for at least 1–2 h, and all measurements were repeated at least three times using different vesicle samples. Proton permeation was initiated by pipetting 1–1.5 μL of an acid solution (1 wt % HCl, 2 wt% HNO_3, 2 wt% HCOOH) to the vesicles sample (60 μL). To capture the initial extravesicular and lumenal pyranine intensities, time-lapse imaging of the vesicles was performed $\approx$1 min prior to the addition of acid. All videos were acquired at a frame rate of either 0.2 frames/s, for HCl and HNO_3, or 0.3 frames/s, for HCOOH.

Image analysis

All images and videos were analyzed using a custom Python script (Zenodo: https://doi.org/10.5281/zenodo.6344232) that automatically detects, tracks, and measures the fluorescence intensity of HPTS within the GUVs lumen. Extraction of vesicles from confocal images (or image stack) was performed through detecting the Liss Rhod PE signal of the GUV equator using the Hough Circle Transform algorithm. Since the sedimented GUVs sometimes move laterally within the image frame boundaries, to ensure a consistent analysis of the same vesicle throughout the duration of the experiment all detected GUVs were tracked and a unique ID was assigned for each one of them. Vesicle tracking was performed by calculating the minimal Euclidean distance between a vesicle's centroid and all other detected centroids in previous frames. Finally, the GUV radius and lumenal HPTS intensity were measured for every image in the acquired measurement video.

RESULTS AND DISCUSSION

Resolving proton permeation across single cell-sized vesicles

Accurate analysis of ion permeation rates from flux measurements across liposomes requires a knowledge of lumenal and extravesicular ion concentrations as well as of their volume and surface area. To this end, we used cell-sized lipid vesicles that can be easily resolved under a light microscope and designed an approach that simultaneously monitors the concentration of ions on both sides of the membrane. Negatively charged GUVs, encompassing a sucrose-containing HEPES buffer solution (buffer A, pH 7.6) and pyranine, a pH-dependent dye that has a negligible membrane permeability during the time course of our experiments, were prepared through a microfluidic-based technique, octanol-assisted liposome assembly (OLA) (37,38). We precisely determined the temporal evolution of proton concentration on both sides of the vesicle membrane by settling GUVs at the bottom of an observation chamber in an isosmotic glucose-containing HEPES buffer (buffer A′, pH 7.6) with a pyranine concentration that matched their interior content (Fig. 1 *A*). Next, a 1–1.5 μL aliquot of acid solution was added to the surrounding solution (see materials and methods) and allowed to diffuse toward and, subsequently, into the vesicles, as schematically illustrated in the upper panel of Fig. 1 *B*.

Through this approach, the fluorescence intensity outside and inside a single GUV can be gradually changed over time (Fig. 1 *B*, *lower panel*) so that the arrival time of protons and their permeation at small concentration gradients can be precisely determined (Fig. 1 *C*), unlike in acid-pulse assays which assume an instantaneous acidification of the external solution (18). We also confirmed the absence of pyranine photobleaching during the measurement period (Fig. S1) and that its fluorescence is altered due to proton permeation and not by leaking through the GUVs membrane (Fig. S2). Furthermore, analysis of extravesicular (*black curve*) and lumenal (*colored curves*) pH values (Figs. 1 *D* and S3) reveals that, following the addition of acid, protons progressively accumulate at the exterior side of the vesicle lipid bilayer while leaking through it at a much slower rate, indicating that their permeation is not rate limited by diffusion through the external unstirred layer (3). Likewise, the fast diffusion time of protons across the internal unstirred layer $t = r^2/6D_{H^+} \approx 2$ ms (i.e., from the membrane to the center of the GUV), relative to the rate of data acquisition in our experiments (see materials and methods), ensures that their concentration inside the vesicles is uniform and measured accurately, where $r = 10$ μm is the vesicle's radius and

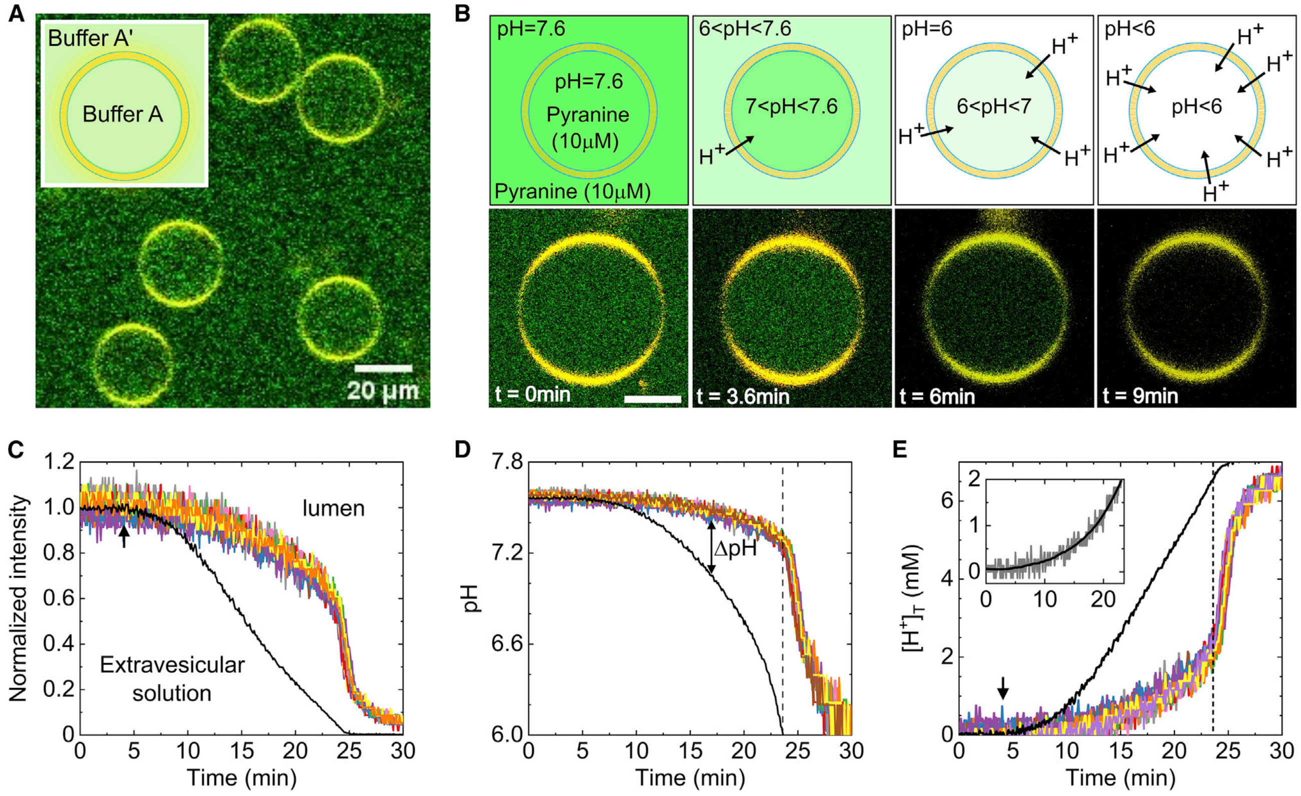

FIGURE 1 Time-resolved analysis of proton permeation across single cell-sized vesicles following the addition of HCl. (*A*) Confocal image of DOPC:DOPG (3:1) GUVs loaded with buffer A (10 mM HEPES, 200 mM sucrose, and 10 μM pyranine; pH 7.6) settled at the bottom of an observation chamber in a buffer A′ (10 mM HEPES, 200 mM glucose, and 10 μM pyranine; pH 7.6). (*B*) Upper: schematic showing the basic concept of the ion permeation assay. A minute volume of acid solution is added to the external buffer and allowed to diffuse toward the resting vesicles, generating a gradually increased pH gradient across their membrane. Lower: proton permeation across a single cell-sized vesicle as illustrated by the change of background and lumenal pyranine intensity. (*C*) Analysis of proton permeation from pyranine fluorescence intensity at the extravesicular solution (*black line*) and lumen of single GUVs (*colored lines*). Permeation measurements were conducted at 21°C. (*D*) Quantification of background and lumenal pH values corresponding to the intensity curves shown in Fig. 2 *C*. The black dashed line signifies the point beyond which the background pH cannot be resolved due to complete quenching of pyranine intensity. (*E*) Total concentration of permeated protons (*colored lines*) and protons outside the vesicles (*black line*). The black dashed line indicates the relevant time frame for flux analysis. Inset: the black line shows an example of a smoothed concentration curve from which the flux is determined. To see this figure in color, go online.

$D_{H^+} = 9.3 \times 10^{-5}$ cm^2/s is the diffusion coefficient of protons (39). Accordingly, the pH difference (ΔpH) across the GUV membrane can be determined for every time frame, allowing us to explore the passive leakage of protons over a range of concentration gradients. In addition, the generation and dissipation rate of ΔpH can be modified by the initial amount of added acid so that the flux of protons can be investigated over a wider range of concentration gradients (Fig. S4). We note, however, that since pyranine is completely quenched below pH = 6 (Fig. S3), proton permeation can only be resolved for $6 < \mathrm{pH} < 7.6$, as indicated by the black dashed line and visualized in Fig. 1 *B*.

Proton permeation under modulated electrochemical gradients

In biological systems, the ionic flux is regulated by the concentration and electric potential gradients across the membrane J(ΔC, Δψ) and is typically described by the Goldman-Hodgkin-Katz (GHK) flux density equation (1):

$$J_{H^+} = P_{H^+} \times \frac{\Delta\psi F}{RT} \times \frac{[H^+]_i - [H^+]_o e^{-\Delta\psi F/RT}}{1 - e^{-\Delta\psi F/RT}},$$

where $[H^+]_i = 10^{-pH_i}/1000$ and $[H^+]_o = 10^{-pH_o}/1000$ are the concentration of H^+ (mol/cm^3) inside and outside the vesicle, respectively, P_{H^+} (cm/s) is the proton permeability coefficient, and *F*, *R*, and *T* have their usual meaning. In the absence of a transmembrane potential, however, the flux density (mol/cm^2/s) depends only on ΔC and is defined by Fick's law:

$$J_{H^+} = P_{H^+} \times \left([H^+]_o - [H^+]_i\right).$$

Remarkably, it has been shown that the conductance (and, thus, the flux) of protons across various lipid bilayers is nearly independent of pH, suggesting that their permeability

coefficient is pH dependent (19,24,40). Therefore, it is of interest to examine the relation between the flux and concentration gradients in our system in order to extract the permeability coefficient and unravel the underlying pathway through which protons passively leak through the lipid bilayer.

By quantifying the concentration of protons in the external solution $[H^+]_o$ from measured pH_o values (Fig. 1 *D*, *black curve*) we are able to determine the concentration gradient of protons $\Delta[H^+] = [H^+]_o - [H^+]_i$ from the time of proton arrival (*black arrow* in Fig. 1 *E*) up to a period where their exterior concentration can no longer be resolved (*black dashed line* in Fig. 1 *E*). The inward flux J_{H^+} (per unit area) of protons was quantified through $J_{H^+} = -\frac{d[H^+]_T^{in}}{dt}\left(\frac{V}{A}\right)$, where *V* is the vesicle volume, *A* is the membrane surface area, and $d[H^+]_T^{in}$ is the change of the total concentration of permeated protons in any time interval d*t*. We analyzed the time-dependant concentration of translocated protons $[H^+]_T^{in}(t) = dpH_i(t) \times \beta(pH)$, shown in Fig. 1 *E* (*colored curves*), through separately measuring the pH-dependent buffer capacity $\beta(pH)$ (Fig. S5) and the variation of lumenal pH (see materials and methods) between consecutive time frames $dpH_i(t) = pH_i(t) - pH_i(t+1)$. Subsequently, the influx of H^+ is found by determining the volume and surface area of each GUV and from the change of $[H^+]_T^{in}$ with respect to time ($d[H^+]_T^{in}/dt$), as determined by smoothing the $[H^+]_T^{in}$ curves (Fig. 1 *E*, *inset*).

Fig. 2 *A* shows the resultant flux profiles $J(\Delta[H^+])$, analyzed from the intensity curves depicted in Fig. 1 *C*. As can be seen, the flux increase saturates as $\Delta[H^+]$ increases, indicating that a transmembrane (diffusion) potential $\Delta\psi$ is gradually accumulating across the GUV membrane (see below). Taking into account that the leakage rate of HCl, in its neutral form, is orders of magnitude higher than that of a proton (H^+) (41) and that at neutral pH HCl is completely dissociated, our result implies that protons cross the membrane predominantly as H^+ and not HCl, as previously suggested to account for their exceptionally high permeability (22,42). On the other hand, at very small values of $\Delta[H^+]$, i.e., when the change of lumenal pH is typically $dpH_i(t) < 0.1$, the flux changes linearly (Fig. 2 *A*, *inset*), implying that $\Delta\psi$ is negligible. Hence, within this linear regime the permeability coefficient of protons can be directly extracted for each vesicle through $P_{H^+} = J_{H^+}/\Delta[H^+]$.

Fig. 2 *B* shows the distribution of P_{H^+} values obtained from different single-vesicle permeation measurements following the addition of HCl. As can be seen, the obtained distribution plot is slightly skewed to the right as only positive P_{H^+} values are possible. Nevertheless, the single peak at $P_{H^+} = 0.002$ cm/s indicates a single population of vesicles with a similar membrane permeability to protons, implying that their translocation mechanism is not dictated by rare processes such as the formation of hydrated (transient) pores in the lipid bilayer, as previously suggested by Kuyper et al. (31). We note, however, that, unlike in the current study, Kuyper et al. examined the permeation of protons across small unilamellar vesicles, adsorbed to a glass surface, by rapidly changing the pH of the external solution from 9 to 3.5—conditions which may impose higher tension on the lipid bilayer and possibly promote pore formation.

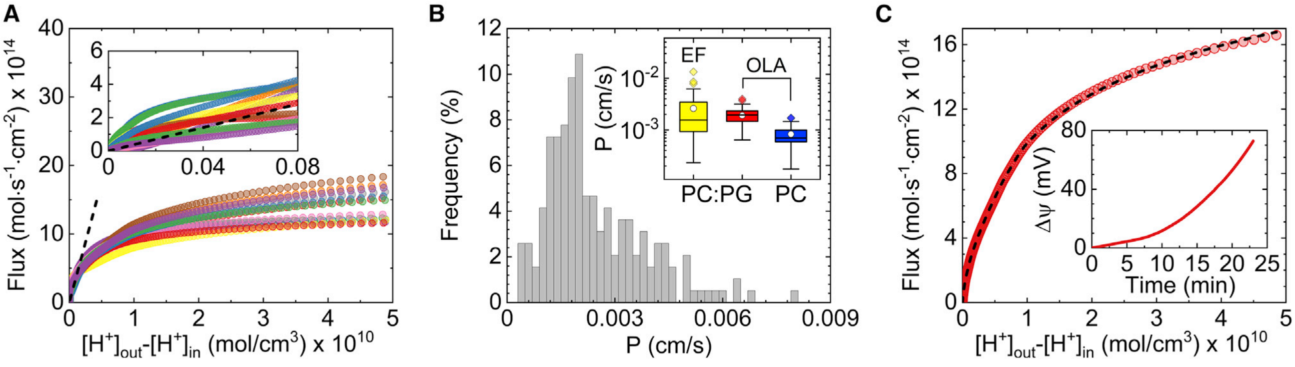

FIGURE 2 Quantification of permeation coefficient and transmembrane potential from flux profiles following the addition of HCl. (*A*) Single-vesicle-level measurement (at 21°C) of proton flux at different concentration gradients, $\Delta[H^+] = [H^+]_o - [H^+]_i$. Inset: a magnified view of the flux profiles at small $\Delta[H^+]$ values color coded to match the curves in the main figure. The black dashed line shown in the inset is the concatenate fit of the flux equation $J_{H^+} = P\Delta[H^+]$ to the plotted flux profiles, as obtained by linear regression. The same linear fit is also shown in the main figure as a black dashed line. (*B*) Frequency distribution of measured permeability coefficients as obtained for negatively charged DOPC:DOPG (3:1 w/w) GUVs ($n = 193$). Inset: standard boxplot depicting the measured proton permeability coefficient P for negatively charged (DOPC:DOPG, 3:1 w/w) electroformed (EF) (*yellow*, $n = 29$) and octanol-assisted liposome assembly (OLA) (*red*, $n = 107$) GUVs and uncharged DOPC OLA-GUVs (*blue*, $n = 25$). The white circles represent the mean. Both PC and PC:PG OLA-GUVs were prepared using the same buffer as in the main figure, but with the addition of 15 vol% glycerol, which was found to have no effect on proton permeability (see supporting material). (*C*) Calculation of transmembrane potential $\Delta\psi$ from a flux profile across a single GUV by solving the GHK equation (see main text) using numerical root finding. The resultant solution is indicated by the black dashed line. Inset: transmembrane potential buildup as extracted from the flux profile shown in the main figure. To see this figure in color, go online.

The obtained permeability coefficients shown in Fig. 2 *B* agree with previously reported P_{H^+} values (10^{-4}–10^{-3} cm/s) (8,18,19,32,43,44). However, it was suggested by Cafiso and Hubbell (45) that P_{H^+} is in fact much smaller, in the range of 10^{-9}–10^{-7} cm/s, as also reported by others (21,22,31), and that larger permeability coefficients may obtain when trace organic contaminants reside in the lipid bilayer. Since OLA-GUVs may possibly contain residual octanol molecules in their membrane, we measured the permeability of protons across electroformed GUVs (Fig. S6), prepared using the same buffer and lipid composition, to examine whether octanol contributes to the large permeability coefficients obtained in our experiments. The inset to Fig. 2 *B* shows the obtained P_{H^+} values following the addition of HCl to electroformed DOPC:DOPG (3:1 w/w) GUVs. As can be seen, the range of obtained values is comparable with that of OLA-GUVs, indicating that proton permeation is not affected by the presence of residual octanol in the membrane.

Another membrane property that may influence the leakage rate of protons in our measurements is the negative surface potential of GUVs (DOPC:DOPG, 3:1). To examine whether P_{H^+} is affected by the surface potential of the lipid bilayer, we prepared uncharged OLA-GUVs that were composed only of DOPC (zwitterionic phospholipid) and measured their permeability to protons following the addition of HCl. Analysis of the obtained flux profiles revealed that the permeation rate of protons across DOPC GUVs is indeed lower than that of negatively charged GUVs prepared under the same experimental conditions (Fig. S7), with average permeability coefficients of $\overline{P}_{H^+}(PC) = 8.3 \times 10^{-4}$ cm/s and $\overline{P}_{H^+}(PC:PG) = 1.9 \times 10^{-3}$ cm/s, respectively (Fig. 2 *B*, *inset*). Furthermore, our finding that $\overline{P}_{H^+}(PC:PG)/\ \overline{P}_{H^+}(PC) \approx 2$ emphasizes the fact that while electrostatic interactions between ions and the lipid bilayer influences their flow across it, the solvation (Born) energy is the dominating energy barrier for ionic leakage across biological membranes (46).

Quantification of electrochemical gradients across single vesicles

We elucidated the influence of $\Delta\psi$ on proton flux by calculating the flux profile of protons in the absence of $\Delta\psi$ using $P_{H^+} = 3.5 \times 10^{-3}$ cm/s as the average permeability coefficient of all flux profiles in the inset to Fig. 2 *A*. A comparison between the expected flux in the absence of electric potential (*black dashed lines*) and the measured flux profiles in Fig. 2 *A* suggests that, in our system, $\Delta\psi$ is significant enough to modulate the flux of protons at small concentration gradients $\Delta[H^+] \sim 0.2 \times 10^{-10}$ mol/cm^3 (= 20 μM). The accumulation of transmembrane potential during proton permeation can be quantified by solving the GHK flux equation for any measured flux profile, using numerical root finding, with $\Delta\psi$ as the only free parameter (all other parameters are measured in the experiment). Fig. 2 *C* demonstrates the solution of the GHK flux model for a flux profile across a single GUV. The resultant diffusion potential (Fig. 2 *C*, *inset*) reveals the gradual buildup of voltage across the membrane as more protons pass through it. Subsequently, the corresponding electrochemical proton gradient, also referred to as the proton motive force (pmf), can be calculated by pmf $= \Delta\psi - 2.3\frac{RT}{F}(pH_i - pH_o)$ to assess the available pmf from passive leakage in our system (47). Likewise, our approach can be utilized to determine the necessary electrochemical gradients for activating membrane proteins, such as ATP synthase and bacterial efflux pumps, in artificial systems such as proteoliposomes.

Fig. 3 shows the evolution of the pmf for the proton flux measurements shown in Fig. 2, with a maximum average value of pmf = 14.2 mV (at T = 21°C) for the average values: $\Delta\psi$ = 45 mV and $\Delta pH \approx 0.53$. The relatively low measured pmf values, compared with bioenergetic membranes (48,49), and appearance of a maximum point in the pmf curve emanate from the presence of two opposing driving forces in our measurements, where ΔpH acts to translocate protons into the vesicle while $\Delta\psi$ acts to move them outside. As such, our measurements suggest that alteration of proton concentration in the external solution may not be sufficient to generate biologically relevant pmf values ($\approx$130–200 mV (48)). Still, in principle, higher pmf values can be achieved through ensuring that both driving forces act in the same direction, for instance, by inducing a negative $\Delta\psi$ with efflux of ions such as K^+, using

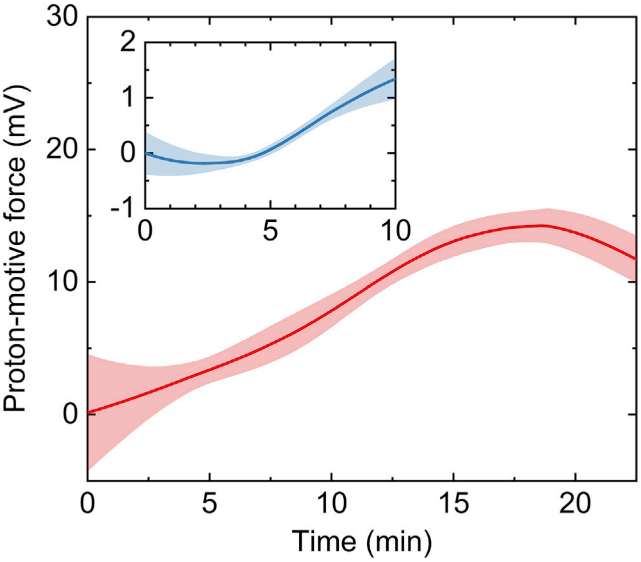

FIGURE 3 Development of the proton motive force (pmf) following the addition of HCl to DOPC:DOPG (3:1 w/w) GUVs (the relevant flux profile is shown in Fig. 2 *A*). Inset: development of the pmf following the addition of formic acid (Fig. 4 *A*). The *solid lines* and *bands* represent the average and standard deviation of the measured pmf, respectively. To see this figure in color, go online.

ionophores like valinomycin, and then acidifying the external solution (7).

Effect of conjugated anion on electrochemical potential generation

Weak organic acids are reported to leak through the lipid bilayer at higher rates than strong inorganic acids, suggesting a distinct translocation route for each type of acid. To elucidate whether electrochemical gradients can be generated using weak acids, we measured the passive proton permeation of formic acid (HCOOH) and compared it with that of two strong acids, HCl and HNO_3. As can already be seen from the intensity plots in Fig. 4 *A*, while the permeation rate of protons appears to be comparable for HCl and HNO_3 it is significantly higher when formic acid is added. The observed difference between weak and strong acids becomes even more evident by analyzing their resultant flux profiles $J(\Delta[H^+])$.

Fig. 4 *B* depicts the flux profiles of protons following the addition of HCl (*black curves*) and HNO_3 (*red curves*). The fact that the HNO_3 profiles saturates at high $\Delta[H^+]$ indicates that, as in the case of HCl, there is a significant buildup of transmembrane potential, since protons mainly cross the membrane as H^+. This strong deviation from linearity is manifested by the clear difference between $J(\Delta[H^+])$ and the calculated proton flux for HNO_3 (Fig. 4 *B*, *black dashed line*) when $\Delta\psi = 0$ mV and the corresponding measured permeability coefficient is $P_{H^+} = (1.1 \pm 0.8) \times 10^{-3}$ cm/s (Fig. 4 *D*). For comparison, the black dotted line in Fig. 4 *B* illustrates the equivalent permeation rate of protons when HCl is added, where $P_{H^+} = (1.7 \pm 1.2) \times 10^{-3}$ cm/s (Fig. 4 *D*). Furthermore, the overlapping flux profiles and diffusion potentials (Fig. 4 *B*, *inset*) of both acids imply that Cl^- and NO_3^- translocate through the membrane at similar rates, as previously mentioned by others (50).

On the contrary, we found that in the case of formic acid, the measured flux varies almost linearly with concentration gradient (Fig. 4 *C*), indicating that the accumulation of charge in the vesicle's interior is significantly lower relative to HCl and HNO_3, as further demonstrated by the difference between the obtained $\Delta\psi$ values for each acid (Fig. 4 *C*, *inset*). Consequently, a very low maximal average electrochemical gradient of pmf = 1.3 mV (at T = 21°C) was

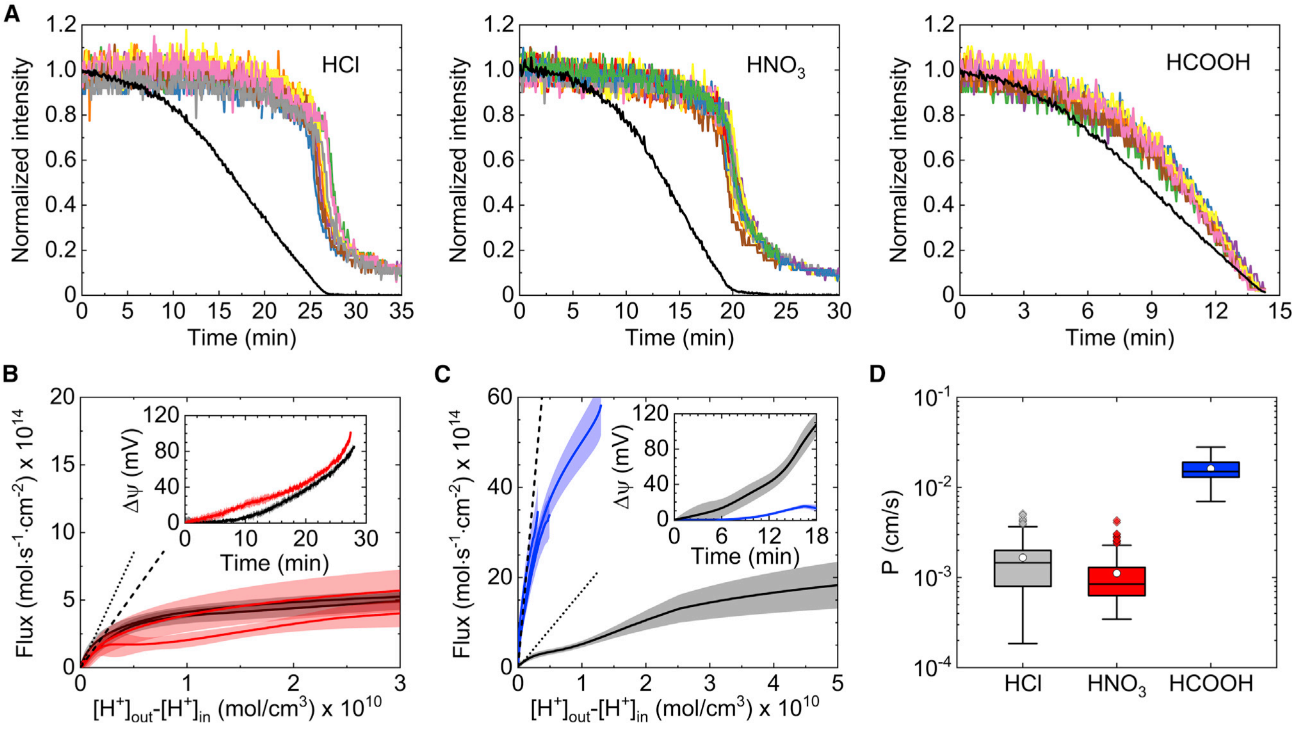

FIGURE 4 Anion-dependent permeability of protons. (*A*) Time-resolved plots of extravesicular (*black line*) and lumenal (*colored lines)* pyranine intensity following the addition of HCl, HNO_3, and formic acid (HCOOH) to DOPC/DOPG (3:1 w/w) GUVs. (*B*) Flux profiles of protons measured for HNO_3 (*red curves*) and HCl (*black curves*). The bold lines and bands represent the average flux profile and standard deviation of all profiles taken for a set of vesicles, respectively. The black dashed and dotted lines are the concatenate fit of the flux equation $J_{H^+} = P\Delta[H^+]$ to the linear regime (not shown) of the plotted HNO_3 and HCl flux profiles, respectively. The comparison between the two acids was repeated twice using different samples of vesicles. Inset: corresponding transmembrane potential buildup for HCl (*black*) and HNO_3 (*red*). (*C*) Flux profiles of protons measured for formic acid (*blue curves*) and HCl (*black curves*). The black dashed and dotted lines are the concatenate fit of the flux equation to the linear regime (not shown) of the plotted HCOOH and HCl flux profiles, respectively. Inset: corresponding transmembrane potential buildup for HCl (*black*) and formic acid (*blue*). (*D*) Standard boxplot of proton permeability coefficients as measured from the flux profiles of HCl ($n = 131$), HNO_3 ($n = 133$), and HCOOH ($n = 21$). The white circles represent the mean *p*-value. To see this figure in color, go online.

generated across the vesicle membrane for the average values: $\Delta\psi = 4.7$ mV, $\Delta pH \approx 0.05$ (Fig. 3, *inset*). The near linear nature of the formic acid flux profile and its variation from the obtained HCl profiles, obtained using the same vesicle sample, are emphasized by the respective dashed and dotted black lines in Fig. 4 *C*. Altogether, permeation analysis provides clear evidence that in the case of formic acid, protons translocate through the lipid bilayer mainly in the form of an uncharged acid (HCOOH) and then dissociate in the vesicle lumen. Accordingly, the permeability coefficient for formic acid $P_{H^+} = (1.6 \pm 0.6) \times 10^{-2}$ cm/s was found to be an order of magnitude larger than P_{H^+} measured for HCl and HNO_3 (Fig. 4 *D*), in agreement with previously reported values (30,51). Similarly, a minimum point appears in the pmf curve of formic acid (Fig. 3, *inset*) due to the rapid development of ΔpH compared with $\Delta\psi$, which rises slowly owing to permeation of undissociated acid molecules.

To verify that proton leakage is indeed governed by a flux of undissociated acid, we quantified the relative portion of permeated HCOOH and H^+ by comparing between the averaged flux profiles of HCl and formic acid at small $\Delta[H^+]$ values, namely, at the linear regime when $\Delta\psi \approx 0$ mV for both acids (Fig. 5). Under the reasonable assumption that only dissociated protons (H^+) cross the membrane when HCl is added (Fig. 2 *A*) and that the permeability rate of the weak acid anion ($HCOO^-$) is a few orders of magnitude smaller than that of HCOOH (17, 28, 29) thus also of H^+, the flux of the undissociated acid (Fig. 5, *red solid line*) can be estimated by $J_{HCOOH} = J_{FA} - J_{H^+}$, where J_{FA} and J_{H^+} are the flux profiles of formic acid (i.e., of both HCOOH and H^+) and HCl, respectively. The relative permeability of HCOOH and H^+ (i.e., the ratio between the slopes of the red and black dotted lines) can then be obtained through $\frac{J_{HCOOH}}{J_{H^+}} = \frac{P_{HCOOH}}{P_{H^+}}$. Here we assume that the flux of HCOOH is governed by $\Delta[H^+]$ since at pH 7.6 the concentration of the undissociated acid is negligible compared with H^+ according to $\frac{[A^-]}{[HA]} = 10^{pH-pK_a} \approx 10^4$. We found that within our experimental conditions $P_{HCOOH}/P_{H^+} = 4.0 \pm 0.1$, meaning that 20% of the protons that traverse into the vesicles lumen cross the membrane as H^+ while the rest permeate as HCOOH. Therefore, while our results show that the governing translocation pathway of weak acids such as HCOOH is via simple diffusion of the undissociated acid, as reported earlier (28–30), the leakage of proton ions and the corresponding accumulation of transmembrane potential cannot be ignored. In addition, since the portion of permeated protons is expected to increase with increasing hydrophilicity of the protonated acid (28,52), our finding indicates that weak acids can be used to generate electrochemical gradients in artificial cell systems.

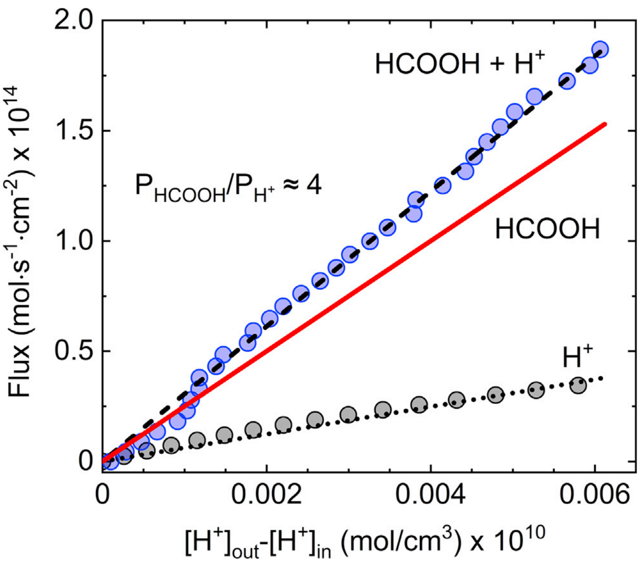

FIGURE 5 Elucidation of formic acid permeation pathway. The gray and blue circles represent the average flux profiles of protons across DOPC/DOPG (3:1 w/w) GUVs following the addition of HCl and formic acid, respectively. The black dashed and dotted lines are the corresponding least-squares fits of $J_{H^+} = P\Delta[H^+]$ to the data with $\overline{P}_{H^+}(HCOOH + H^+) = 3.1 \times 10^{-2} \pm 1.8 \times 10^{-4}$ cm/s and $\overline{P}_{H^+}(HCl) = 6.2 \times 10^{-3} \pm 1.2 \times 10^{-4}$ cm/s. The red solid line is the flux profile of the undissociated formic acid, estimated through subtracting the black dotted line (flux of H^+) from the black dashed line (flux of HCOOH + H^+). The slope of the red curve is the evaluated permeability coefficient of formic acid, $\overline{P}_{HCOOH} = 2.5 \times 10^{-2} \pm 2.2 \times 10^{-4}$ cm/s. To see this figure in color, go online.

CONCLUSIONS

To conclude, we directly quantified the permeation coefficient and flux of protons across single cell-sized lipid vesicles under modulated electrochemical gradients. Through analyzing the obtained flux profiles, we determined the accumulation of transmembrane potential (i.e., level of membrane polarization) as a result of proton permeation following the addition of different types of acids. Our results reveal that in the case of a weak acid (formic acid), protons accumulate in the vesicle interior owing to the synchronous permeation of uncharged acid molecules (AH) and protons (H^+), while in the case of strong acids (HCl or HNO_3) proton permeation was governed by translocation of H^+. Accordingly, a larger pmf was obtained for strong acids (pmf = 14.2 mV) relative to formic acid (pmf = 1.3 mV). Further analysis of proton flux, following the addition of formic acid, revealed that out of the total amount of permeated protons, a fraction of ≈ 0.2 traverses the membrane as H^+, with the rest (≈ 0.8) in the form of a neutral acid. As such we show that, using our approach, the correlation between proton permeation and pmf can be elucidated for different types of acids. We anticipate that this approach can be employed for resolving protein-based transport of protons using biomimetic giant vesicles and molecular dynamic simulations and will guide future efforts to unravel the membrane permeation of vital acids, including fatty acids and carboxylic acid-containing drugs.

SUPPORTING MATERIAL

Supporting material can be found online at https://doi.org/10.1016/j.bpj.2022.05.041.

AUTHOR CONTRIBUTIONS

R.T. designed the experiments, conducted the measurements, analyzed the data, and wrote the manuscript. M.F. assisted with coding. M.F. and U.F.K. assisted with theoretical aspects of ion transport. All authors discussed the results and commented on the manuscript.

ACKNOWLEDGMENTS

R.T. acknowledges funding from the European Union's Horizon 2020 research and innovation program under the Marie Sklodowska-Curie grant agreement no. 892333, and from the Blavatnik Family Foundation. U.F.K. and R.T. acknowledge support from an ERC consolidator grant (Designer-Pores 647144). M.F. acknowledges support from the UK's Engineering and Physical Sciences Research Council Doctoral Training Programme (EP/R513180/1) and a Cambridge-NPL case studentship.

DECLARATION OF INTERESTS

The authors declare no competing interests.

REFERENCES

1. Stein, W. 2012. Transport and Diffusion across Cell Membranes. Elsevier.
2. Gadsby, D. C. 2009. Ion channels versus ion pumps: the principal difference, in principle. *Nat. Rev. Mol. Cell Biol.* 10:344–352. https://doi.org/10.1038/nrm2668.
3. Hannesschlaeger, C., A. Horner, and P. Pohl. 2019. Intrinsic membrane permeability to small molecules. *Chem. Rev.* 119:5922–5953. https://doi.org/10.1021/acs.chemrev.8b00560.
4. Stein, W. D., and T. Litman. 2014. Channels, Carriers, and Pumps: An Introduction to Membrane Transport. Elsevier.
5. Yanagisawa, M., M. Iwamoto, …, S. Oiki. 2011. Oriented reconstitution of a membrane protein in a giant unilamellar vesicle: experimental verification with the potassium channel KcsA. *J. Am. Chem. Soc.* 133:11774–11779. https://doi.org/10.1021/ja2040859.
6. Lee, K. Y., S.-J. Park, …, K. Shin. 2018. Photosynthetic artificial organelles sustain and control ATP-dependent reactions in a protocellular system. *Nat. Biotechnol.* 36:530–535. https://doi.org/10.1038/nbt.4140.
7. Turina, P., D. Samoray, and P. Gräber. 2003. H+/ATP ratio of proton transport-coupled ATP synthesis and hydrolysis catalysed by CF0F1—liposomes. *EMBO J.* 22:418–426. https://doi.org/10.1093/emboj/cdg073.
8. Veshaguri, S., S. M. Christensen, …, D. Stamou. 2016. Direct observation of proton pumping by a eukaryotic P-type ATPase. *Science.* 351:1469–1473. https://doi.org/10.1126/science.aad6429.
9. Garni, M., T. Einfalt, …, W. Meier. 2018. Live follow-up of enzymatic reactions inside the cavities of synthetic giant unilamellar vesicles equipped with membrane proteins mimicking cell architecture. *ACS Synth. Biol.* 7:2116–2125. https://doi.org/10.1021/acssynbio.8b00104.
10. Ghadiri, M. R., J. R. Granja, and L. K. Buehler. 1994. Artificial transmembrane ion channels from self-assembling peptide nanotubes. *Nature.* 369:301–304. https://doi.org/10.1038/369301a0.
11. Tunuguntla, R. H., F. I. Allen, …, A. Noy. 2016. Ultrafast proton transport in sub-1-nm diameter carbon nanotube porins. *Nat. Nanotechnol.* 11:639–644. https://doi.org/10.1038/nnano.2016.43.
12. Pick, H., A. C. Alves, and H. Vogel. 2018. Single-vesicle assays using liposomes and cell-derived vesicles: from modeling complex membrane processes to synthetic biology and biomedical applications. *Chem. Rev.* 118:8598–8654. https://doi.org/10.1021/acs.chemrev.7b00777.
13. Pagano, R., and T. Thompson. 1968. Spherical lipid bilayer membranes: electrical and isotopic studies of ion permeability. *J. Mol. Biol.* 38:41–57. https://doi.org/10.1016/0022-2836(68)90127-7.
14. Toyoshima, Y., and T. E. Thompson. 1975. Chloride flux in bilayer membranes. Chloride permeability in aqueous dispersions of single-walled, bilayer vesicles. *Biochemistry.* 14:1525–1531. https://doi.org/10.1021/bi00678a028.
15. Paula, S., A. Volkov, and D. Deamer. 1998. Permeation of halide anions through phospholipid bilayers occurs by the solubility-diffusion mechanism. *Biophys. J.* 74:319–327. https://doi.org/10.1016/s0006-3495(98)77789-6.
16. Paula, S., A. Volkov, …, D. W. Deamer. 1996. Permeation of protons, potassium ions, and small polar molecules through phospholipid bilayers as a function of membrane thickness. *Biophys. J.* 70:339–348. https://doi.org/10.1016/s0006-3495(96)79575-9.
17. Gabba, M., J. Frallicciardi, …, B. Poolman. 2020. Weak acid permeation in synthetic lipid vesicles and across the yeast plasma membrane. *Biophys. J.* 118:422–434. https://doi.org/10.1016/j.bpj.2019.11.3384.
18. Deamer, D. W., and J. W. Nichols. 1983. Proton-hydroxide permeability of liposomes. *Proc. Natl. Acad. Sci. U S A.* 80:165–168. https://doi.org/10.1073/pnas.80.1.165.
19. Nichols, J. W., and D. W. Deamer. 1980. Net proton-hydroxyl permeability of large unilamellar liposomes measured by an acid-base titration technique. *Proc. Natl. Acad. Sci. U S A.* 77:2038–2042. https://doi.org/10.1073/pnas.77.4.2038.
20. Wylie Nichols, J., M. W. Hill, …, D. W. Deamer. 1980. Measurement of net proton-hydroxyl permeability of large unilamellar liposomes with the fluorescent pH probe, 9-aminoacridine. *Biochim. Biophys. Acta Biomembr.* 596:393–403. https://doi.org/10.1016/0005-2736(80)90126-1.
21. Perkins, W. R., and D. S. Cafiso. 1986. An electrical and structural characterization of proton/hydroxide currents in phospholipid vesicles. *Biochemistry.* 25:2270–2276. https://doi.org/10.1021/bi00356a063.
22. Nozaki, Y., and C. Tanford. 1981. Proton and hydroxide ion permeability of phospholipid vesicles. *Proc. Natl. Acad. Sci. U S A.* 78:4324–4328. https://doi.org/10.1073/pnas.78.7.4324.
23. Deamer, D. W. 1987. Proton permeation of lipid bilayers. *J. Bioenerg. Biomembr.* 19:457–479. https://doi.org/10.1007/BF00770030.
24. Gutknecht, J. 1987. Proton/hydroxide conductance and permeability through phospholipid bilayer membranes. *Proc. Natl. Acad. Sci. U S A.* 84:6443–6446. https://doi.org/10.1073/pnas.84.18.6443.
25. Tepper, H. L., and G. A. Voth. 2006. Mechanisms of passive ion permeation through lipid bilayers: insights from simulations. *J. Phys. Chem. B.* 110:21327–21337. https://doi.org/10.1021/jp064192h.
26. Zhang, H.-Y., Q. Xu, …, D.-Q. Wei. 2016. Passive transmembrane permeation mechanisms of monovalent ions explored by molecular dynamics simulations. *J. Chem. Theor. Comput.* 12:4959–4969. https://doi.org/10.1021/acs.jctc.6b00695.
27. Dezi, M., A. Di Cicco, …, D. Lévy. 2013. Detergent-mediated incorporation of transmembrane proteins in giant unilamellar vesicles with controlled physiological contents. *Proc. Natl. Acad. Sci. U S A.* 110:7276–7281. https://doi.org/10.1073/pnas.1303857110.
28. Saparov, S. M., Y. N. Antonenko, and P. Pohl. 2006. A new model of weak acid permeation through membranes revisited: does Overton still rule? *Biophys. J.* 90:L86–L88. https://doi.org/10.1529/biophysj.106.084343.
29. Gutknecht, J., and D. C. Tosteson. 1973. Diffusion of weak acids across lipid bilayer membranes: effects of chemical reactions in the unstirred

layers. *Science.* 182:1258–1261. https://doi.org/10.1126/science.182.4118.1258.

30. Walter, A., and J. Gutknecht. 1984. Monocarboxylic acid permeation through lipid bilayer membranes. *J. Membr. Biol.* 77:255–264. https://doi.org/10.1007/bf01870573.
31. Kuyper, C. L., J. S. Kuo, …, D. T. Chiu. 2006. Proton permeation into single vesicles occurs via a sequential two-step mechanism and is heterogeneous. *J. Am. Chem. Soc.* 128:3233–3240. https://doi.org/10.1021/ja057349c.
32. Guha, A., M. L. McGuire, …, M. Mayer. 2021. A single-liposome assay that enables temperature-dependent measurement of proton permeability of extremophile-inspired lipid membranes. *Biochim. Biophys. Acta Biomembr.* 1863:183567. https://doi.org/10.1016/j.bbamem.2021.183567.
33. Walde, P., K. Cosentino, …, P. Stano. 2010. Giant vesicles: preparations and applications. *Chembiochem.* 11:848–865. https://doi.org/10.1002/cbic.201000010.
34. Li, S., P. Hu, and N. Malmstadt. 2011. Imaging molecular transport across lipid bilayers. *Biophys. J.* 101:700–708. https://doi.org/10.1016/j.bpj.2011.06.044.
35. Bąk, K. M., B. van Kolck, …, M. J. Chmielewski. 2020. Oxyanion transport across lipid bilayers: direct measurements in large and giant unilamellar vesicles. *Chem. Commun.* 56:4910–4913. https://doi.org/10.1039/c9cc09888g.
36. Valkenier, H., N. López Mora, …, A. P. Davis. 2015. Visualization and quantification of transmembrane ion transport into giant unilamellar vesicles. *Angew. Chem. Int. Ed.* 127:2165–2169. https://doi.org/10.1002/ange.201410200.
37. Tivony, R., M. Fletcher, …, U. F. Keyser. 2021. A microfluidic platform for sequential assembly and separation of synthetic cell models. *ACS Synth. Biol.* 10:3105–3116. https://doi.org/10.1021/acssynbio.1c00371.
38. Deshpande, S., Y. Caspi, …, C. Dekker. 2016. Octanol-assisted liposome assembly on chip. *Nat. Commun.* 7:1–9.
39. Agmon, N. 1995. The grotthuss mechanism. *Chem. Phys. Lett.* 244:456–462. https://doi.org/10.1016/0009-2614(95)00905-j.
40. Gutknecht, J. 1987. Proton conductance through phospholipid bilayers: water wires or weak acids? *J. Bioenerg. Biomembr.* 19:427–442. https://doi.org/10.1007/BF00770028.
41. Khramtsov, V., M. Panteleev, and L. Weiner. 1989. ESR study of proton transport across phospholipid vesicle membranes. *J. Biochem. Biophys. Methods.* 18:237–246. https://doi.org/10.1016/0165-022x(89)90008-0.
42. Gutknecht, J., and A. Walter. 1981. Transport of protons and hydrochloric acid through lipid bilayer membranes. *Biochim. Biophys. Acta Biomembr.* 641:183–188. https://doi.org/10.1016/0005-2736(81)90582-4.
43. Biegel, C. M., and J. M. Gould. 1981. Kinetics of hydrogen ion diffusion across phospholipid vesicle membranes. *Biochemistry.* 20:3474–3479. https://doi.org/10.1021/bi00515a026.
44. Rossignol, M., P. Thomas, and C. Grignon. 1982. Proton permeability of liposomes from natural phospholipid mixtures. *Biochim. Biophys. Acta Biomembr.* 684:195–199. https://doi.org/10.1016/0005-2736(82)90005-0.
45. Cafiso, D. S., and W. L. Hubbell. 1983. Electrogenic H+/OH-movement across phospholipid vesicles measured by spin-labeled hydrophobic ions. *Biophys. J.* 44:49–57. https://doi.org/10.1016/s0006-3495(83)84276-3.
46. Parsegian, A. 1969. Energy of an ion crossing a low dielectric membrane: solutions to four relevant electrostatic problems. *Nature.* 221:844–846. https://doi.org/10.1038/221844a0.
47. Grabe, M., and G. Oster. 2001. Regulation of organelle acidity. *J. Gen. Physiol.* 117:329–344. https://doi.org/10.1085/jgp.117.4.329.
48. Calisto, F., F. M. Sousa, …, M. M. Pereira. 2021. Mechanisms of energy transduction by charge translocating membrane proteins. *Chem. Rev.* 121:1804–1844. https://doi.org/10.1021/acs.chemrev.0c00830.
49. Brzezinski, P., A. Moe, and P. Ädelroth. 2021. Structure and mechanism of respiratory III–IV supercomplexes in bioenergetic membranes. *Chem. Rev.* 121:9644–9673. https://doi.org/10.1021/acs.chemrev.1c00140.
50. Samouilov, A., Y. Y. Woldman, …, V. Khramtsov. 2007. Magnetic resonance study of the transmembrane nitrite diffusion. *Nitric Oxide.* 16:362–370. https://doi.org/10.1016/j.niox.2006.12.006.
51. Walter, A., and J. Gutknecht. 1986. Permeability of small nonelectrolytes through lipid bilayer membranes. *J. Membr. Biol.* 90:207–217. https://doi.org/10.1007/bf01870127.
52. Missner, A., P. Kügler, …, P. Pohl. 2008. Passive transport across bilayer lipid membranes: overton continues to rule. *Proc. Natl. Acad. Sci. U S A.* 105:E123. https://doi.org/10.1073/pnas.0809606106.

Biophysical Journal, Volume *121*

Supplemental information

Quantifying proton-induced membrane polarization in single biomimetic giant vesicles

Ran Tivony, Marcus Fletcher, and Ulrich F. Keyser

Supplementary information for:

Quantifying proton-induced membrane polarization in single biomimetic giant vesicles

Ran Tivony*, Marcus Fletcher and Ulrich F Keyser

Cavendish Laboratory, University of Cambridge, JJ Thomson Avenue, Cambridge CB3 0HE, UK.

* Corresponding author: E-mail: rt497@cam.ac.uk

Table of content

S1. Photobleaching of pyranine 2

S2. Verifying the absence of pyranine leakage during permeation measurements 2

S3. pH-dependent variation of pyranine intensity 3

S4. The effect of added acid amount on dissipation rate of proton gradients 4

S5. Buffer capacity 5

S6. Proton permeation across negatively charged electroformed GUVs (DOPC:DOPG) 6

S7. The effect of lipid-bilayer charge on proton permeability 6

S1. Photobleaching of pyranine

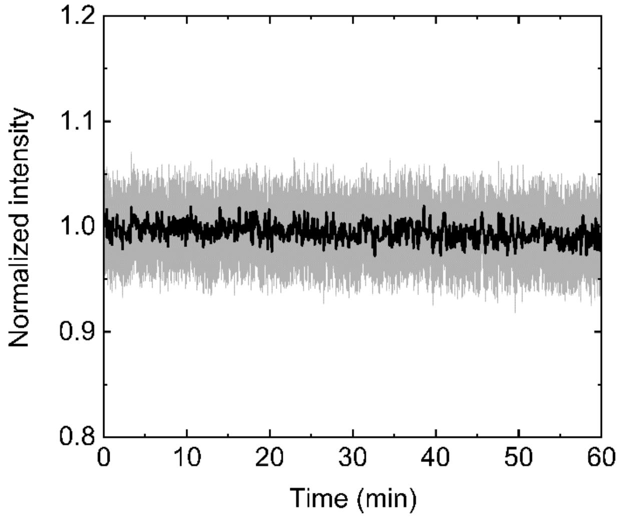

Figure S1. Photobleaching of pyranine. The normalized intensity of pyranine, encapsulated within the GUVs lumen, was measured through a confocal microscope using the same acquisition rate (0.2 frames/sec) and laser power as in proton permeation experiments shown in the main paper. The black line and gray band are the average normalized intensity and standard deviation of all profiles taken for a set of vesicles (n = 89).

S2. Verifying the absence of pyranine leakage during permeation measurements

To accurately quantify the inward flux of protons we verified that the measured reduction of pyranine intensity is due to proton permeation and not as a result of pyranine leakage out of the vesicles. For this purpose, we first added HCl to initiate the leakage of protons inside the GUVs and then (after ~10min) we increased the pH of the external solution by adding 1µl of 1M NaOH. Therefore, in case that the observed drop in fluorescence signal, following acidification with HCl, is indeed due to pyranine leakage out of the vesicles, no change in pyranine intensity is expected to occur upon raising the pH value of the GUVs interior. Figure S4A shows the gradual decrease of pyranine intensity as a result of decreasing the pH outside of the vesicles, where the black trace is the extravesicular solution and each coloured trace signifies the lumen of a single GUV. As can be seen in figure S4B, upon adding NaOH, the

fluorescence intensity inside the vesicles (coloured traces) progressively increases towards the intensity of the of the external solution (black trace), indicating that pyranine remained encapsulated in the GUVs during proton permeation.

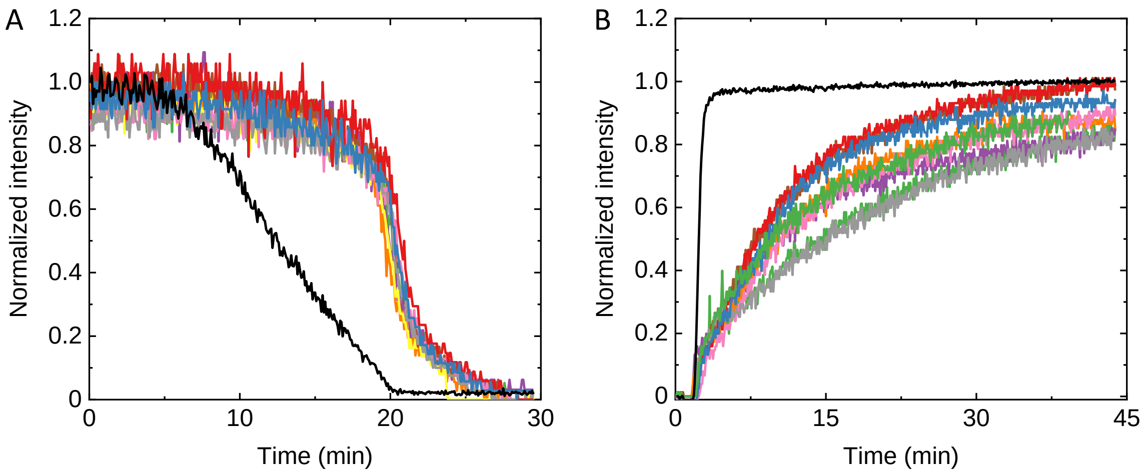

Figure S2. Pyranine fluorescence intensity inside GUVs following acidification (A) and basification (B) of the external solution. The black and coloured traces signify the intensity of the extravesicular solution and GUVs lumen, where each trace in A and B is colour-coded to match the same GUV.

S3. <u>pH-dependent variation of pyranine intensity</u>

To quantify the pH from intensity data, we measured the intensity of pyranine (HPTS) at different pH values and under the same conditions in which the permeation experiments were conducted (figure S1). Fluorescence measurements were taken by a confocal microscope and pyranine was dissolved in buffer A' (see main text) to a final concentration of 10µM. Consequently, the pH value the pH inside and outside every detected GUVs was calculated from the obtained pyranine intensity (I) using, $\mathrm{pH} = 0.53 \ln(\mathrm{I}) + 7.65$.

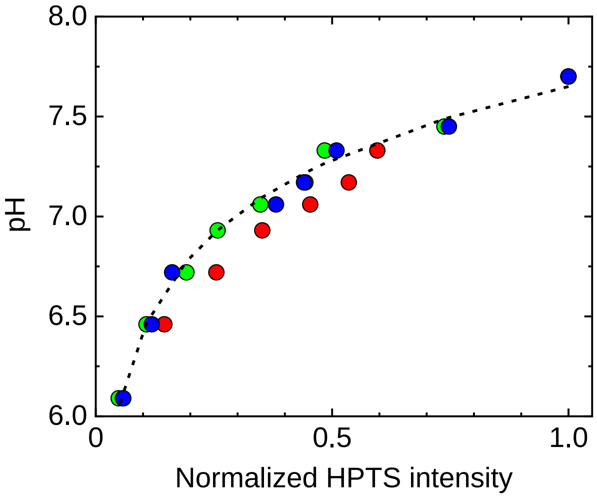

Figure S3. pH-dependent intensity of pyranine. Each symbol represent a separate measurement and the black dashed line represents the best fit to data using least squares regression.

S4. The effect of added acid amount on dissipation rate of proton gradients

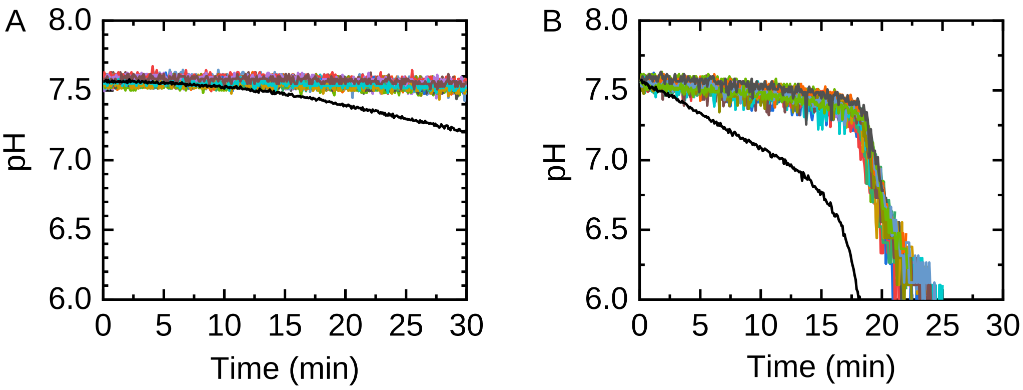

Figure S4. Quantification of lumenal (coloured lines) and extravesicular (black lines) pH values following the addition of 1µl (A) and 1.5µl (B) 1% HCl to the GUVs solution (60µl). The corresponding acid concentrations in the GUVs solution are 5.4mM and 8mM, respectively.

S5. Buffer capacity

The buffer capacity, β, indicates the efficiency of a buffer to resist its change of pH following an addition of n moles of protons ($\beta = dn/dpH$). Since buffer capacity depends on the pH value of the buffer, we measured $\beta(pH)$ by titrating the buffers used in this study (see experimental section) with known amounts of protons (dn), and analyzed the resultant pH change (dpH) at various pH values. The obtained $\beta(pH)$ curves were fitted using a polynomial equation to calculate the buffer capacity at any measured lumenal pH. The concentration of protons in the GUVs interior was then extracted using the following relation $[H^+]_i = \beta(pH) \times dpH/V$, where V is the vesicles volume.

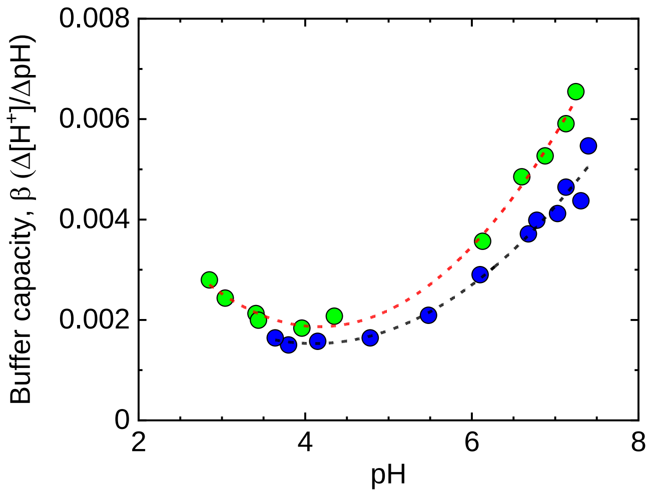

Figure S5. The blue and green circles are the $\beta(pH)$ values measured for buffer A' with and without 15% v/v glycerol. The and red and black dashed line are the best fit of a polynomial to the data using least squares. The following equations were obtained for the red and black fits: $\beta = 0.0005 \times pH^2 - 0.004 \times pH + 0.0102$ and $\beta = 0.0003 \times pH^2 - 0.0027 \times pH + 0.007$, respectively.

S6. Proton permeation across negatively charged electroformed GUVs (DOPC:DOPG)

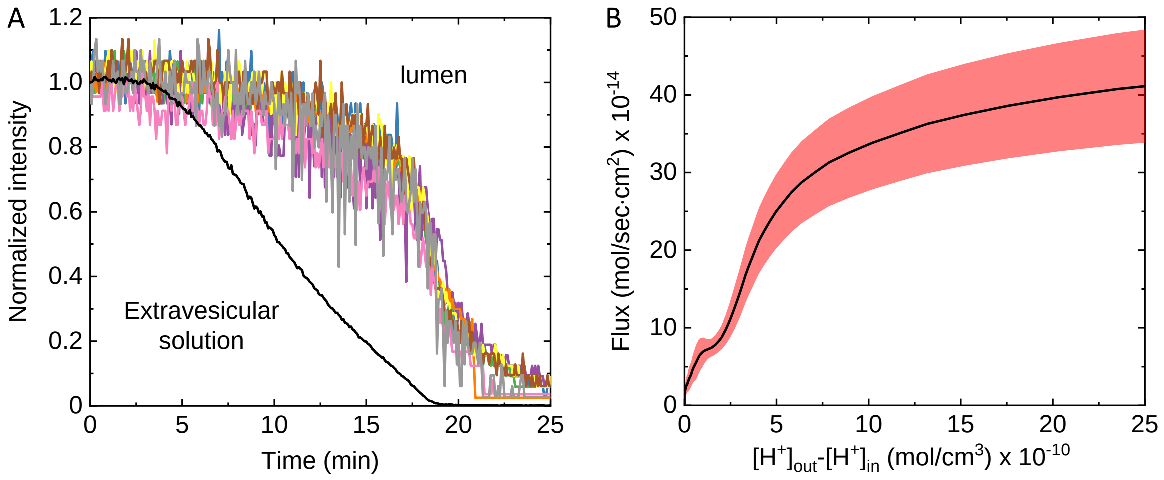

Figure S6. Proton permeation measurement across electroformed GUVs (DOPC:DOPG, 3:1 w/w) under the same conditions and buffer used for proton permeation measurement using OLA GUVs (see experimental data). **A.** Time-resolved fluorescence intensity of pyranine following the addition of HCl. The black curve indicates the intensity change outside the vesicles and the coloured curves indicates the intensity change of the lumen, where each curve represents a single GUV. **B.** Flux profiles of protons measured across the GUVs depicted in figure S6A. The bold black line and red band represent the average flux profile and standard deviation of all profiles taken for a set of vesicles, respectively.

S7. The effect of lipid-bilayer charge on proton permeability

To examine the effect of surface charge on the leakage rate of protons P_{H^+}, we prepared uncharged GUVs from the zwitterionic phospholipid DOPC (1,2-dioleoyl-sn-glycero-3-phosphocholine) and measured their permeability to protons following the addition of HCl. Since DOPC GUVs were found to be less stable than negatively charged DOPC:DOPG GUVs in the buffer solution used in our experiments (see experimental section), we used the same buffer but with glycerol (buffer A + 15% v/v glycerol). To confirm that glycerol does not affect the permeability of the lipid bilayer, we prepared DOPC:DOPG GUVs in 15% glycerol buffer and compared the obtained flux profile and permeability coefficient of protons (figure S5A,

red curves) to that of DOPC:DOPG GUVs that were prepare in the same buffer but without glycerol (figure S5B).

As can be seen, similar flux profiles and permeability coefficients (inset to figure S5B) were obtained in the presence and absence of glycerol, indicating that, at this concentration, glycerol does not influence the passive leakage of protons through the vesicles membrane. On the other hand, the flux of protons across DOPC GUVs (figure S5A, blue curves) was found to be lower than that of negatively charged vesicles. In addition, lower permeability coefficients were measured for the uncharged vesicles ($\overline{P}_{H^+}(PC) = 8.3 \times 10^{-4}$ cm/s vs. $\overline{P}_{H^+}(PC:PG) = 1.9 \times 10^{-3}$ cm/s) as demonstrated by the linear flux profiles shown in the inset to figure S5A. Altogether, our results suggest that the membrane permeability to protons (and most likely for other ions) is affected, though not dramatically, by the surface charge of the lipid vesicles.

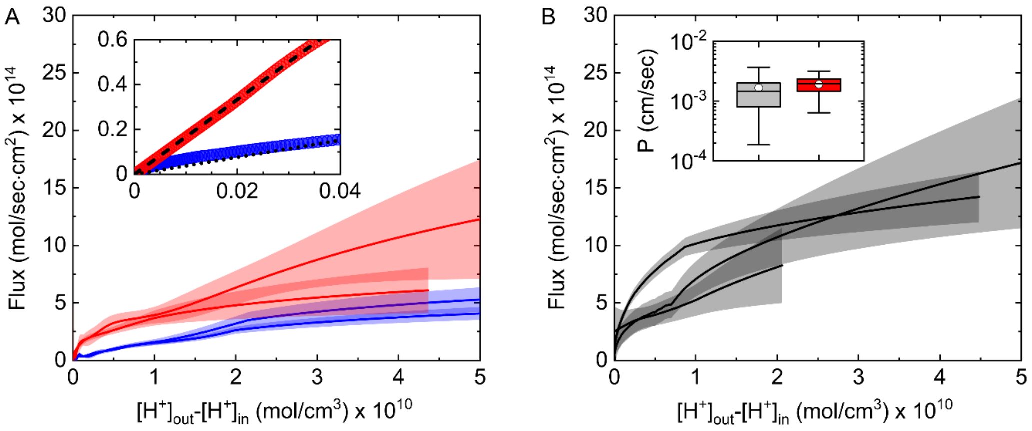

Figure S7. Influence of membrane charge on proton permeability. **A.** Flux profiles of protons measured in 15% glycerol buffer for DOPC:DOPG negatively charge GUVs (red curves) and DOPC uncharged GUVs (blue curves) following the addition of HCl. The bold lines and bands represent the average flux profile and standard deviation of all profiles taken for a set of vesicles. The comparison between the two types of vesicles was repeated twice using different samples of vesicles. Inset: average flux profiles of protons at small concentration gradients colour-coded to match the flux curves in the main figure. **B.** Flux profiles of protons measured in buffer A (no glycerol) for DOPC:DOPG negatively charge GUVs following the addition of HCl. Inset: standard box plot of proton permeability coefficients in the absence (gray) and presence (red) of 15% v/v glycerol.
